# The T3SS Effector EspT Defines a New Category of Invasive Enteropathogenic *E. coli* (EPEC) Which Form Intracellular Actin Pedestals

**DOI:** 10.1371/journal.ppat.1000683

**Published:** 2009-12-11

**Authors:** Richard Bulgin, Ana Arbeloa, David Goulding, Gordon Dougan, Valerie F. Crepin, Benoit Raymond, Gad Frankel

**Affiliations:** 1 Centre for Molecular Microbiology and Infection, Division of Cell and Molecular Biology, Imperial College London, London, United Kingdom; 2 The Wellcome Trust Sanger Institute, Wellcome Trust Genome Campus, Hinxton, Cambridge, United Kingdom; Institut Pasteur, France

## Abstract

Enteropathogenic *Escherichia coli* (EPEC) strains are defined as extracellular pathogens which nucleate actin rich pedestal-like membrane extensions on intestinal enterocytes to which they intimately adhere. EPEC infection is mediated by type III secretion system effectors, which modulate host cell signaling. Recently we have shown that the WxxxE effector EspT activates Rac1 and Cdc42 leading to formation of membrane ruffles and lamellipodia. Here we report that EspT-induced membrane ruffles facilitate EPEC invasion into non-phagocytic cells in a process involving Rac1 and Wave2. Internalized EPEC resides within a vacuole and Tir is localized to the vacuolar membrane, resulting in actin polymerization and formation of intracellular pedestals. To the best of our knowledge this is the first time a pathogen has been shown to induce formation of actin comets across a vacuole membrane. Moreover, our data breaks the dogma of EPEC as an extracellular pathogen and defines a new category of invasive EPEC.

## Author Summary

Enteropathogenic *E. coli* (EPEC) is an important diarrheal pathogen responsible for significant infant mortality in the developing world and is increasingly associated with sporadic outbreaks in the developed world. The virulence strategy of EPEC revolves around a conserved Type 3 secretion system (T3SS) which translocates bacterial effector proteins directly into host cells. EPEC is considered to be a non-invasive pathogen which intimately adheres to host cells and polymerizes actin rich pedestals on which extracellular bacteria rest. Recently we have identified the T3SS effector EspT which activates the mammalian Rho GTPases Rac1 and Cdc42, resulting in the formation of membrane ruffles and lamellipodia. In this study we dissect the signaling pathway utilized by EspT to nucleate membrane ruffles and demonstrate that these ruffles can promote EPEC invasion of host cells. Furthermore, we show that internalized EPEC are bound within a vacuole. We also report for the first time the ability of a bacterial pathogen to form actin comet tails across a vacuole membrane. In addition to providing novel insights into the subversion of cellular signaling by invasive pathogens, our study also breaks the long held dogma of EPEC as an extracellular pathogen and will have implications on how future EPEC infections are diagnosed and treated.

## Introduction

The human pathogens enteropathogenic *Escherichia coli* (EPEC) and enterohemorrhagic *E. coli* (EHEC) [1] and the mouse pathogen *Citrobacter rodentium* [2] are closely related extra-cellular diarrhoeal agents characterized by their ability to colonize the gut epithelium via attaching and effacing (A/E) lesion formation (reviewed in [3]) [4]. Similarly to other Gram-negative bacteria EPEC, EHEC and *C. rodentium* encode a type III secretion system (T3SS), which is central to their infection strategy (reviewed in [5]) [6]. This complex machinery translocate dozens of effector proteins [7],[8] directly from the bacteria to the eukaryotic cell cytoplasm (reviewed in [9]). The translocated effectors are targeted to various sub-cellular compartments where they subvert a plethora of cell signaling pathways via interactions with a range of host cell proteins.

The host cell cytoskeleton is a common target of T3SS effectors [10]. EPEC, EHEC and *C. rodentium* translocate the effector Tir into the plasma membrane where it functions as a receptor for the bacterial outer membrane protein intimin [11]. Intimin:Tir interaction leads to activation of N-WASP and formation of actin rich pedestals on which the extracellular bacteria rest [12]. In addition to Tir, A/E pathogens translocate a variety of other effectors which also modulate the host cell cytoskeleton including EspG/EspG2, which induce depolymerization of the microtubule network [13], Map, which induces formation of transient filopodia early in infection [14] and EspM which directs formation of actin stress fibers [15]. Map and EspM are members of the WxxxE family [15],[16],[17], which was first grouped together based on conserved peptide motif consisting of an invariant tryptophan and glutamic acid residues separated by three variable amino acids and their shared ability to subvert host cell small GTPase signaling.

Small GTPases cycle between an inactive GDP bound and an active GTP bound form, allowing them to function as molecular switches in response to a variety of stimuli. The switch from inactive to active forms results in a conformational change, which allows the GTPase to bind downstream mammalian effectors. Small GTPases are regulated by guanine exchange factors (GEFs), GTPase activating proteins (GAPs) and guanine dissociation inhibitor (GDI) proteins (reviewed in [18],[19]). The three best characterized Rho GTPases are RhoA, Rac1 and Cdc42 which are implicated in formation of stress fibers, lamellipodia and filopodia respectively (reviewed in [20]).

The WxxxE effectors were originally proposed to be functional mimics of mammalian small GTPases [16]. However, we have recently shown that EspM activates RhoA [15] whereas Map induces filopodia via activation of Cdc42 and RhoA [17]. In addition to Map and EspM we have recently discovered the novel WxxxE effector EspT, which is encoded by *C. rodentium* and a subset of EPEC strains [21], including EPEC E110019 which caused a sever outbreak in Finland in 1987 that affected children and adults alike [22]. We have shown that EspT induces formation of lamellipodia and membrane ruffles in epithelial cells via activation of Rac1 and Cdc42 [23].

Membrane ruffles are sheet like structures which are induced by mammalian cells in order to facilitate crawling movement, macro-pinocitosis and receptor recycling (reviewed in [24]). These protrusion are regulated through activity of Rho family GTPases and their downstream effectors (reviewed in [25]). Importantly, a subset of invasive bacterial pathogens hijack and subvert mammalian signal transduction pathways which facilitate formation membrane ruffles in order to promote bacterial entry into mammalian cells. Perhaps the best studied of these pathogens are *Salmonella* and *Shigella* which induce extensive membrane ruffles at the site of bacterial attachment (reviewed in [26],[27]). *Salmonella* invasion is dependent upon the activity of several T3SS effector proteins including SopE/E2 which act as GEFs for Rac1 and Cdc42 [28] and SopB which activates the RhoG GEF SGEF [29]. *Shigella* has also evolved several invasive mechanisms. For example the translocator IpaC has been shown to induce ruffles at the site of *Shigella* entry via the activation of Cdc42 [30], recruitment of Src kinase [31] and activation of Abl kinase [32]. The *Shigella* WxxxE effector IpgB1 has also been shown to induce membrane ruffles via interaction with the ELMO DOCK180 complex which results in activation of Rac1 [33].

EPEC, EHEC and *C. rodentium* are generally considered extracellular pathogens and their attachment sites on epithelial cells are normally characterized by the assembly of an actin rich pedestal rather than membrane ruffles (reviewed in [3]). However, in both rabbit and human biopsies EPEC have been visualized inside enterocytes and detected in the sub mucosa, mesenteric lymph nodes and spleen [34] (reviewed in [35]). Recently Hernandes *et al* has shown that the atypical EPEC strain 1551-2 is capable of invading cultured epithelial cells in an intimin omicron dependent manner [36]. As EspT induces membrane ruffles similar to those triggered by IpgB1 [37] the aim of this study was to investigate if expression of EspT leads to EPEC cell invasion and to define the underlying mechanism.

## Results

### EspT-induced membrane ruffles surround adherent bacteria

A large screen of clinical EPEC isolates for the presence of *espT*, a T3SS effector (Fig. S1), has shown that the gene is present in ca. 1.8% of the tested strains [21]. In order to investigate the role of EspT in cell invasion we selected to use the *espT* positive strains E110019 and *C. rodentum*; the *espT* negative EPEC, strain JPN15 [38], was used as a control. In addition, we generated a JPN15 clone that expresses EspT from the bacterial expression vector pSA10 (pICC461).

**Figure 1:**
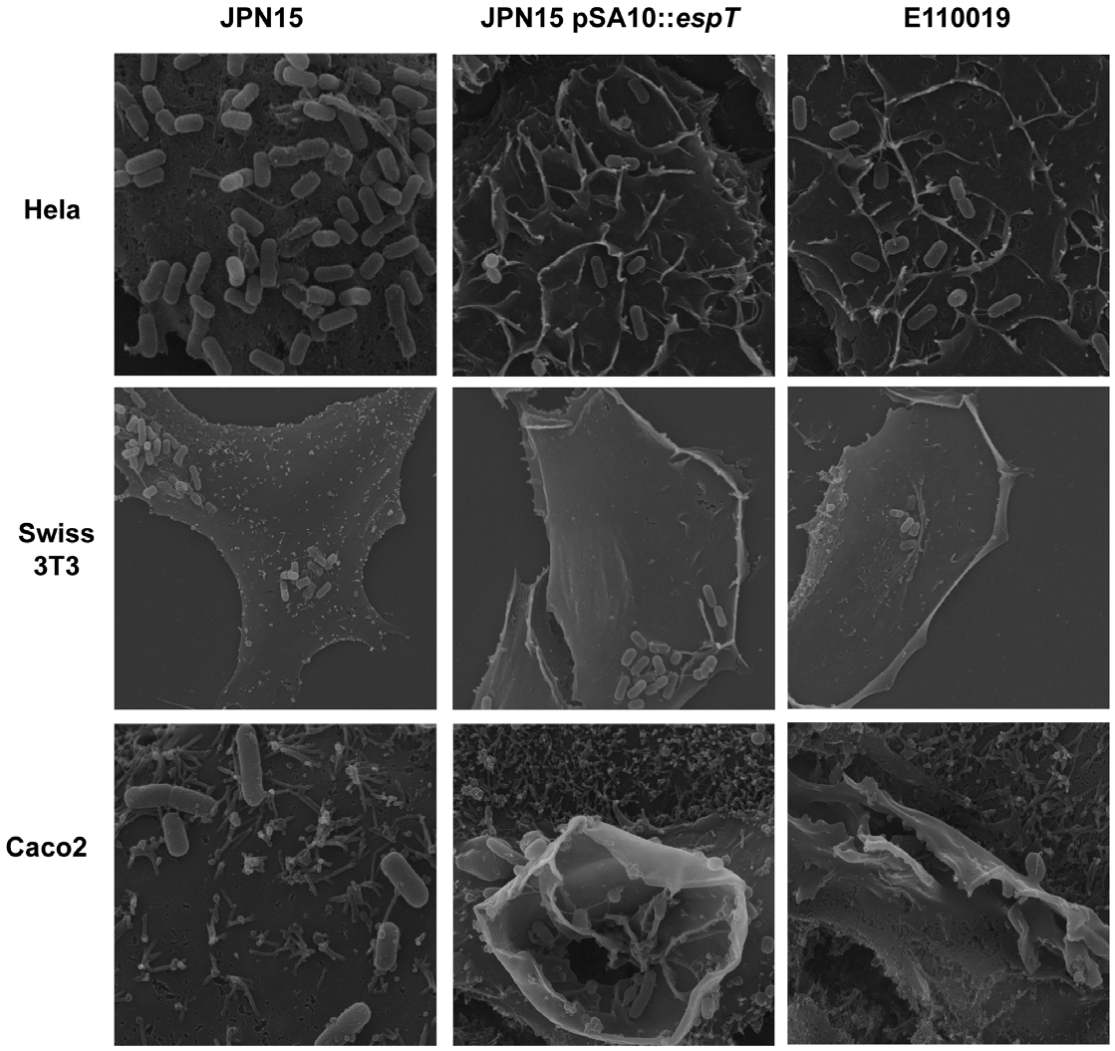
Scanning electron microscopy of HeLa, Swiss 3T3 and polarized Caco2 cells infected with JPN15, JPN15 expressing EspT or E110019 for 2 h. JPN15 displayed a pattern of diffused adherence on HeLa, Swiss 3T3 and Caco2 cells but did not induce any significant membrane remodeling. JPN15 expressing EspT and E110019 also adhered to HeLa, Swiss 3T3 and Caco2 cells in a diffuse pattern but induced membrane ruffles at the site of bacterial attachment, which were more pronounced on Caco2 cells. In addition to membrane ruffles JPN15 expressing EspT and E110019 also induced formation of lamellipodia and dorsal ruffles at locations distal from the site of bacterial attachment on Swiss 3T3 cells. Magnifications: HeLa cells X5000; Swiss 3T3 X3500; Caco2 X10000 and X6500 (middle).

We infected serum starved HeLa, Swiss 3T3 and Caco2 cells with E110019, JPN15 and JPN15 expressing EspT; the cells were then fixed and processed for scanning electron microscopy (SEM). The JPN15-infected HeLa and Swiss 3T3 cells displayed characteristic diffuse bacterial adhesion without any noteworthy surface structures. Caco2 cells infected with JPN15 also show a diffuse pattern of bacterial adherence and a concordant localized effacement of microvili (Fig. 1). HeLa cells infected with JPN15 expressing EspT or E110019 displayed extensive membrane ruffling over the entire cell surface (Fig. 1); in the vicinity of adherent bacteria the ruffles surrounded and wrapped individual bacterial cells forming structures which appear permissive for internalization. Swiss 3T3 cells infected with JPN15 expressing EspT or E110019 exhibited extensive dorsal ruffles and lamellipodia in addition to localized membrane ruffles at the site of bacterial attachment (Fig. 1). Caco2 cells infected with JPN15 expressing EspT or E110019 displayed prominent membrane ruffles at the site of bacterial adherence in addition to effacement of micovili (Fig. 1). These results show that EspT can induce actin remodeling and surface structures, similar to those associated with *Shigella* and *Salmonella* invasion (reviewed in [26]).

### Wave2 and Abi1 are localized to membrane ruffles and lamellipodia induced by EspT

**Figure 2:**
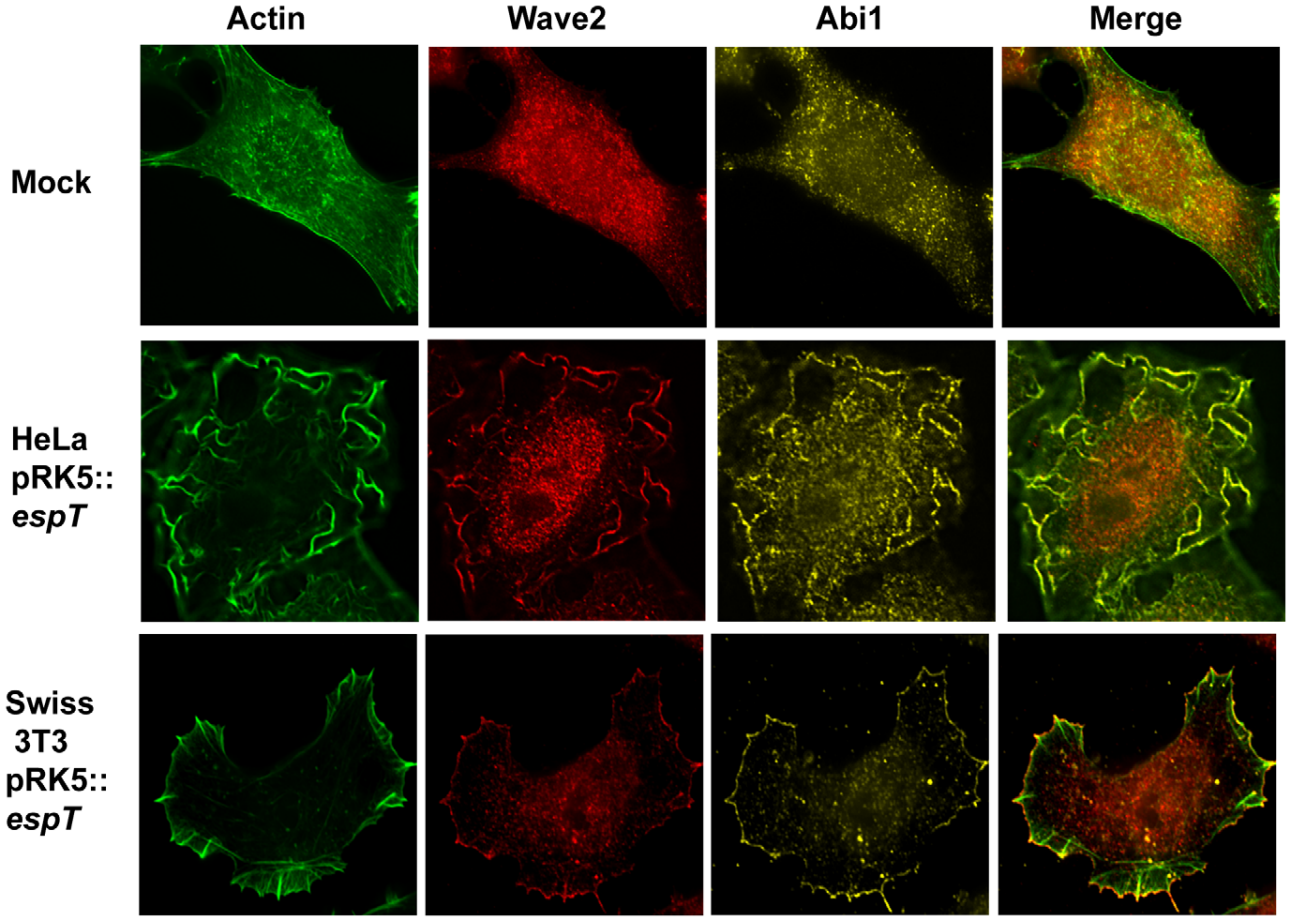
Wave2 and Abi1 are localized to membrane ruffles and lamellipodia induced by ectopically expressed EspT. Actin was stained with Oregon Green phalloidin (Green), Wave2 was detected with a polyclonal rabbit antibody (Red) and Abi1 was visualized using a mouse monoclonal antibody (Yellow). Mock transfected cells did not display any significant actin structures and Wave2 and Abi1 were localized diffusely in the cytoplasm. HeLa cells transfected with pRK5::*espT* exhibit prominent membrane ruffles on their apical surface which were enriched with Wave2 and Abi1. Ectopic expression of EspT in Swiss 3T3 cells resulted in formation of distinctive lamellipodia to which Wave2 and Abi1 were extensively recruited and co-localized.

We have recently shown that remodeling of the host cell actin cytoskeleton by EspT is dependent on Rac1 and to a lesser extent Cdc42 [23]. Rac1 and Cdc42 utilize a plethora of downstream effectors in order to regulate cytoskeletal dynamics (reviewed in [19] and [25]). Several GTPase effectors including IRSp53, N-WASP, Pak, Wave2 and Abi1 have been previously been implicated in formation of membrane ruffles [39],[40],[41],[42]. By using immuno-fluorescence microscopy we found that both Wave2 and Abi1 were present and co-localized with actin at membrane ruffles and the leading edge of lamellipodia induced by EspT (Fig. 2), while N-WASP was not (data not shown). The signaling protein IRSp53 has been proposed to participate in Abi1-Wave2-Rac1 complex formation [39],[43]. While we did not detect any significant enrichment of IRSp53 in lamellipodia, IRSp53 was localized to membrane ruffles nucleated by EspT (Fig. S2). Taken together these results show that Abi1 and Wave2 are localized to membrane ruffles and lamellipodia induced by EspT but IRSp53 is only recruited to EspT-induced membrane ruffles.

### Wave2 is essential for EspT-induced membrane remodeling

**Figure 3:**
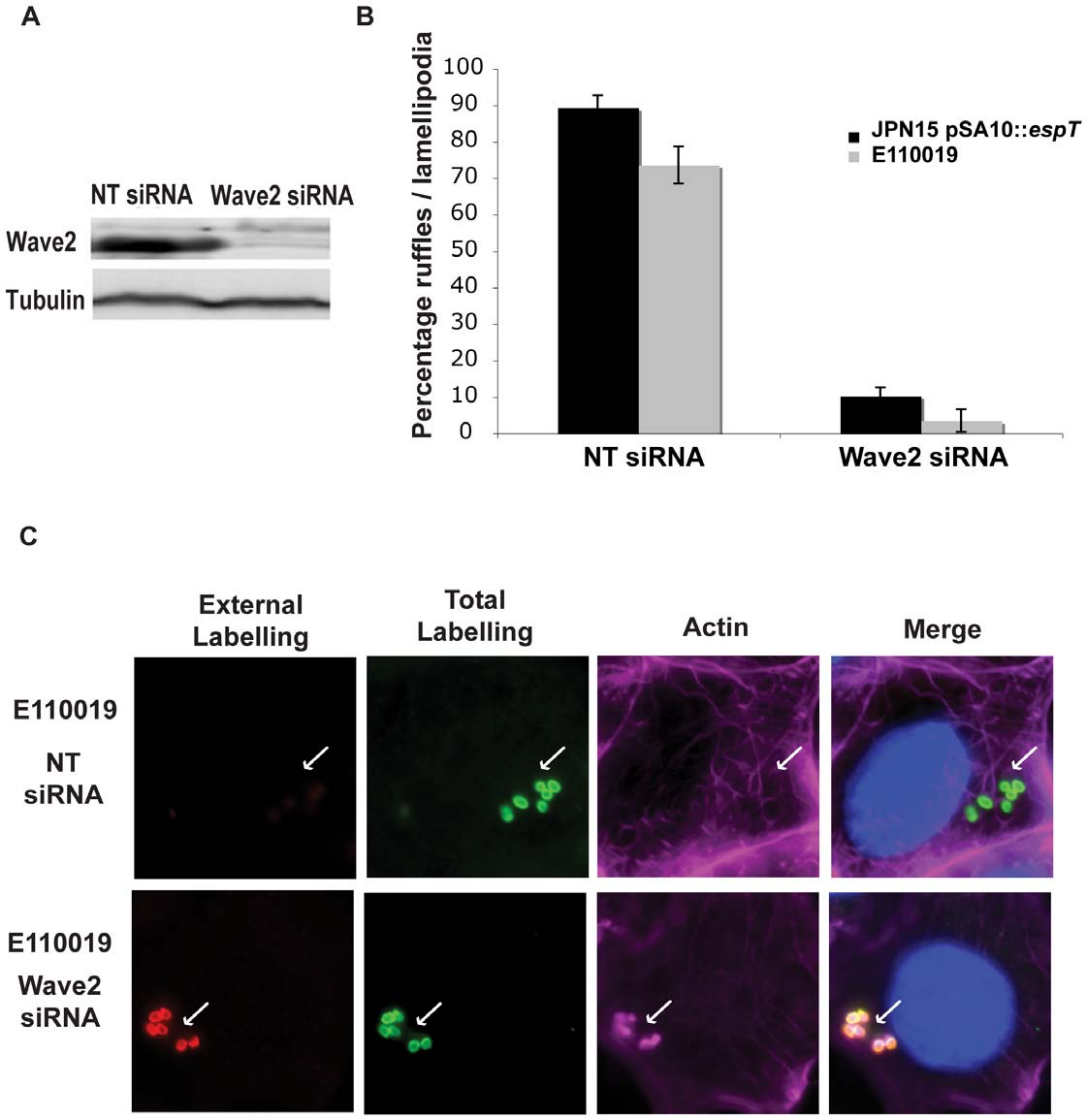
Wave2 is essential for EspT induced membrane remodeling and invasion. (A) Swiss 3T3 cells were treated with Non Targeting (NT) siRNA or siRNA targeted against Wave2. (A) Western blot with lysates from Swiss 3T3 cells treated with NT and Wave2 siRNA. Wave2 and Tubulin were detected with monoclonal antibodies. Non Targeting siRNA did not alter Wave2 expression whereas treatment with Wave2 siRNA depleted the protein. Protein levels in the lysates were normalized using anti tubulin antibodies. (B) Quantification of membrane remodeling induced by E110019 and JPN15 expressing EspT in Swiss 3T3 cells treated with NT and Wave2 siRNA after 3 h of infection. 100 cells were counted in triplicate resulted are presented as mean±SEM. Treatment with Wave2 siRNA oligos reduced the level of membrane remodeling induced by JPN15 pSA10::*espT* and E110019 to 10% and 5% respectively. This is comparable to the 9% of cells which display membrane ruffle and lamellipodia formation in cells infected with EPEC not expressing EspT. (C) Cells were infected with E110019 for 3 h were fixed and stained prior to permeabilization (extracellular labeling) (Red). The cells were then washed, permeabilized, re-labeled (Total labeling) (Green) along with Alexaflour 633 Phalloidin (Cyan) and Dapi (Blue). In cells treated with the NT siRNA E110019 induced formation of membrane ruffles and a large proportion of bacteria were labeled by the total stain which were absent from the extracellular labeling. Depletion of Wave2 using siRNA inhibited formation of membrane ruffles by E110019 and the majority of bacteria were detected by both the extracellular and total staining demonstrating that depletion of Wave2 inhibits EPEC invasion.

Wave2 is a ubiquitously expressed member of the WASP super family of actin regulators which potently activates the Arp2/3 complex [44]. The Wave family of proteins have a modular structure consisting of a N terminal Wave homology domain (WHD), a central proline rich region (PRR) and a C terminal Arp2/3 binding domain (VCA module) (reviewed in [45]). The WHD domain has been shown to bind Abi1 [42] and the PRR has been shown to interact with the SH3 domain of IRSp53 [39]. We utilized siRNA in order to determine if Wave2 is essential for formation of the EspT-dependent membrane ruffles. Depletion of endogenous Wave2 from Swiss 3T3 cells, confirmed by Western blotting (Fig. 3A), resulted in a marked decrease in formation of membrane ruffles and lamellipodia induced by JPN15 expressing EspT or E110019, compared with cells treated with scrambled siRNA (Fig. 3B). In order to determine which of the Wave2 domains is required for formation of lamellipodia and membrane ruffles by EspT, we transfected Swiss 3T3 and HeLa cells with full length Wave2 or dominant negative forms of Wave2 lacking the WHD (ΔBP) or the acidic Arp2/3 interacting domain (ΔA). Transfected cell were infected for 1.5 h with JPN15 expressing EspT and the presence of lamellipodia or membrane ruffles was assessed. Mock transfected cells or cells transfected with full length Wave2 had lamellipodia and membrane ruffles on 80 to 90% of infected cells (Fig. S3). In contrast, transfection with either the ΔBP or the ΔA Wave2 dominant negative constructs resulted in significant reduction in lamellipodia and membrane ruffle formation (Fig. S3). This result demonstrates that binding of Arp2/3 to Wave2 is essential for EspT-mediated formation of lamellipodia and membrane ruffles. Furthermore, the observation that the N terminal truncated Wave2 ΔBP construct has a dominant negative effect suggests that the WHD motif is also required for EspT mediated cytoskeletal rearrangements. The fact that the Wave2 ΔBP construct, which is capable of binding IRSp53 but not Abi1, is not sufficient to induce EspT dependent actin remodeling further indicates that IRSp53 does not play a prominent role in EspT mediated signaling.

### EspT facilitates EPEC invasion

Induction of membrane ruffles is a mechanism employed by a range of pathogenic bacteria in order to facilitate cell invasion. This method of bacterial invasion is referred to as the trigger mechanism and relies upon induction of actin polymerization to form an entry foci and a macropinocytic pocket (reviewed in [26]). JPN15 expressing EspT and E110019 induce host cell membrane remodeling which is reminiscent of entry foci and membrane ruffles induced by *Shigella* and *Salmonella* (reviewed in [26]) (Fig. 1).

We used differential staining to visualize invasion of Swiss 3T3 cells by JPN15, JPN15 expressing EspT, and E110019; *Salmonella enterica* serovar Typhimurium strain SL1344 was used as a control. In addition, we conducted gentamycin protection assays to quantify cell invasion of Swiss 3T3, HeLa and Caco2 cells after 3 h infection. Differential immuno-fluorescence staining and gentamycin protection assays were also performed in HeLa and Swiss 3T3 cells infected for 6 h with wild type *C. rodentium*, *C. rodentium* Δ*espT* and complemented *C. rodentium* Δ*espT*. For immuno-fluorescence extracellular bacteria were stained pre cell permeabilzation with primary anti O127 (JPN15), anti O111 (E110019), anti O152 (*C. rodentium*) or anti LPS (*S.* Typhimurium) antibodies and a secondary antibody coupled to a Cy3 fluorophore (red). The cells were then permeabilized and total bacteria were stained with the same primary antibodies and a secondary antibody coupled to a Cy2 fluorophore (green), Alexafluor 633 phalloidin and Dapi were used to visualize actin and DNA respectively.

**Figure 4:**
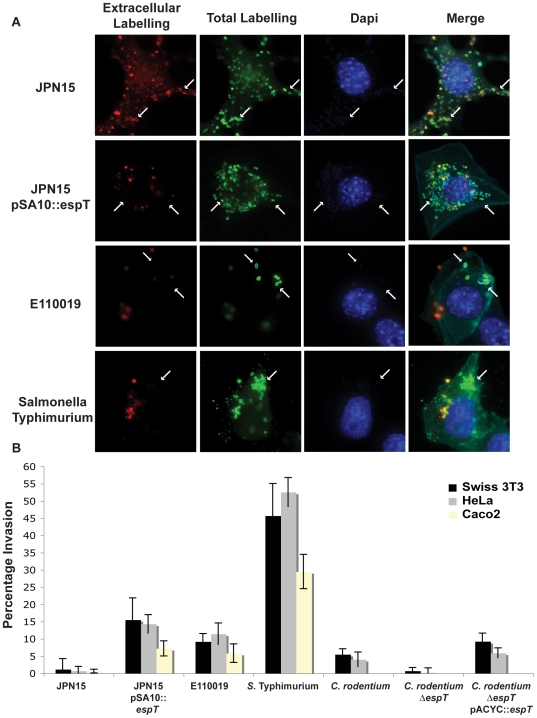
EspT-dependent actin remodeling facilitates bacterial invasion of epithelial cells. (A) Swiss 3T3 cells infected with JPN15, JPN15 expressing EspT, E110019 or *S.* Typhimurium for 3 h were fixed and stained prior to permeabilization (extracellular labeling) (Red). The cells were then washed, permeabilized, re-labeled (Total labeling) (Green) along with Alexaflour 633 Phalloidin (Cyan) and Dapi (Blue). In cells infected with JPN15 all the bacteria labeled by the total staining were also detected with the extracellular probe indicating that there was no significant invasion (highlighted with arrows). Significant numbers of bacteria labeled with the total stain, which were not represented in the extracellular staining, were seen in cells infected JPN15 expressing EspT, E110019 and *S.* Typhimurium indicating that there was a significant degree of bacterial invasion (highlighted with arrows). (B) Gentamycin protection assay of Swiss 3T3 and HeLa cells infected JPN15, JPN15 expressing EspT, E110019, *S.* Typhimurium, *C. rodentium*, *C. rodentium* Δ*espT* or complemented *C. rodentium* Δ*espT* and polarized Caco2 cells infected with JPN15, JPN15 expressing EspT, E110019 or *S.* Typhimurium. Results are representative of 3 independent experiments carried out in duplicate and are displayed as mean±SEM.

Adherent JPN15 bacteria were homogenously stained by both the extracellular and total bacterial probes, indicating that this strain is not significantly invasive (Fig. 4A). In cells infected with JPN15 expressing EspT or E110019 a significant proportion of the bacteria were labeled with the total bacterial stain but not by the extracellular probe (Fig. 4A). *S.* Typhimurium-infected cells exhibited characteristic membrane ruffling at the entry foci and a high proportion of bacteria were labeled only with the total bacterial probe (Fig. 4A).

The quantitative gentamycin protection assay revealed that JPN15 does not efficiently invade HeLa, Swiss 3T3 or Caco2 cells, exhibiting an invasion rate of less than 1.5% (Fig. 4B). JPN15 expressing EspT was significantly more invasive with an invasion rate of 15.5% in Swiss 3T3, 14.3% in HeLa and 7.2% in Caco2 cells (Fig. 4B). E110019 invaded Swiss 3T3, HeLa and Caco2 cells at a rate of 9.2%, 11.4% and 5.8% respectively. The invasive capacity of EPEC was significantly less than *S.* Typhimuriumin (Fig. 4B). Infection of HeLa cells with JPN15 expressing EspT^W63A^ for 3 h confirmed that the WxxxE motif plays a major role in membrane ruffling and cell invasion (Fig. S4).

E110019 is multi drug resistant, which limits the ability to genetically modified the isolate. In order to determine if cell invasion is mediated by EspT, we infected Swiss 3T3 cells for 6 h with wild type *C. rodentium* and *C. rodentium* Δ*espT*. Infection with wild type *C. rodentium* resulted in membrane ruffles and cell invasion, while the *espT* mutant exhibited neither (Fig. 4B). Complementing the mutant with *espT* expressed from pACYC184 (pICC489) restored membrane ruffle formation and cell invasion (Fig. 4B and S5).

In order to confirm that EspT can promote EPEC invasion of non-phagocytic cells independently of other T3SS effectors we ectopically expressed EspT in HeLa cells prior to infection with EPEC Δ*escN*, a T3SS null mutant. Cells ectopically expressing EspT displayed membrane ruffling which facilitated the uptake of Δ*escN* bacteria (Fig. S6). No membrane ruffles were observed in cells ectopically expressing EspTW63A (data not shown). These results show that EspT induces EPEC cell invasion by a trigger mechanism, analogous to that of *Shigella* and *Salmonella*.

### Rac1 and Wave2 are essential for EspT mediated bacterial cell invasion

**Figure 5:**
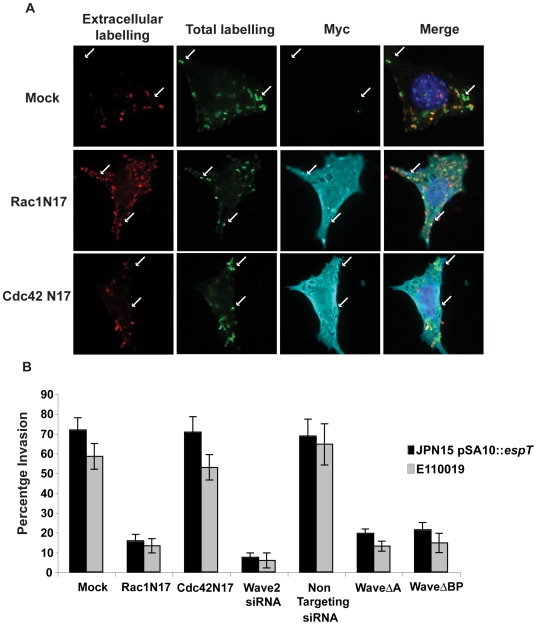
Rac1 and Wave2 are essential for EspT-mediated bacterial invasion. (A) Swiss 3T3 cells were left untransfected or transfected with Non Targeting (NT) or Wave2 siRNA olgos, the ectopic expression vector pDSRed encoding Wave2ΔA, Wave2ΔBP or pRK5 expressing dominant negative Rac1 and Cdc42 and infected with JPN15, JPN15 expressing EspT or E110019. Mock transfected cells or cells transfected with dominant negative Cdc42 were efficiently invaded by both JPN15 expressing EspT and E110019. Cells transfected with dominant negative Rac1 were significantly more resistant to bacterial invasion. The Wave2ΔA and Wave2ΔBP constructs also had a potent dominant negative effect on bacterial internalization. (B) Quantification of bacterial invasion; cells which had 3 or more internalized bacteria were scored as invaded. 100 cells were counted in triplicate in three independent experiments. Results are displayed as mean±SEM.

As actin remodeling by EspT is dependent on activation of Rac1, Cdc42 [23] and Wave2 (Fig. 3), we utilized dominant negative constructs of these signaling proteins and Wave2 siRNA to monitor the effect on invasion of JPN15 expressing EspT and E110019. Swiss 3T3 cells transfected with dominant negative Rac1 (Rac1^N17^), Cdc42 (Cdc42^N17^), Wave2ΔA truncated in the acidic Arp2/3 interacting region and Wave2ΔBP lacking the WHD were infected for 3 h. The cells were fixed and stained for bacterial invasion as described above. Cells transfected with Cdc42^N17^ were still permissive of bacterial invasion while cells transfected with the Rac1^N17^, Wave2ΔA or Wave2ΔBP dominant negative constructs were not (Fig. 5B). Depletion of Wave2 using siRNA in Swiss 3T3 cells significantly reduced the invasive capacity of both JPN15 expressing EspT and E110019 compared to cells treated with non-targeting siRNA (Fig. 3A and 5B). Thus, Rac1, Wave2 and Abi1 are essential mediators of EspT-induced bacterial invasion.

### Internalized EPEC are bound within a vacuole

**Figure 6:**
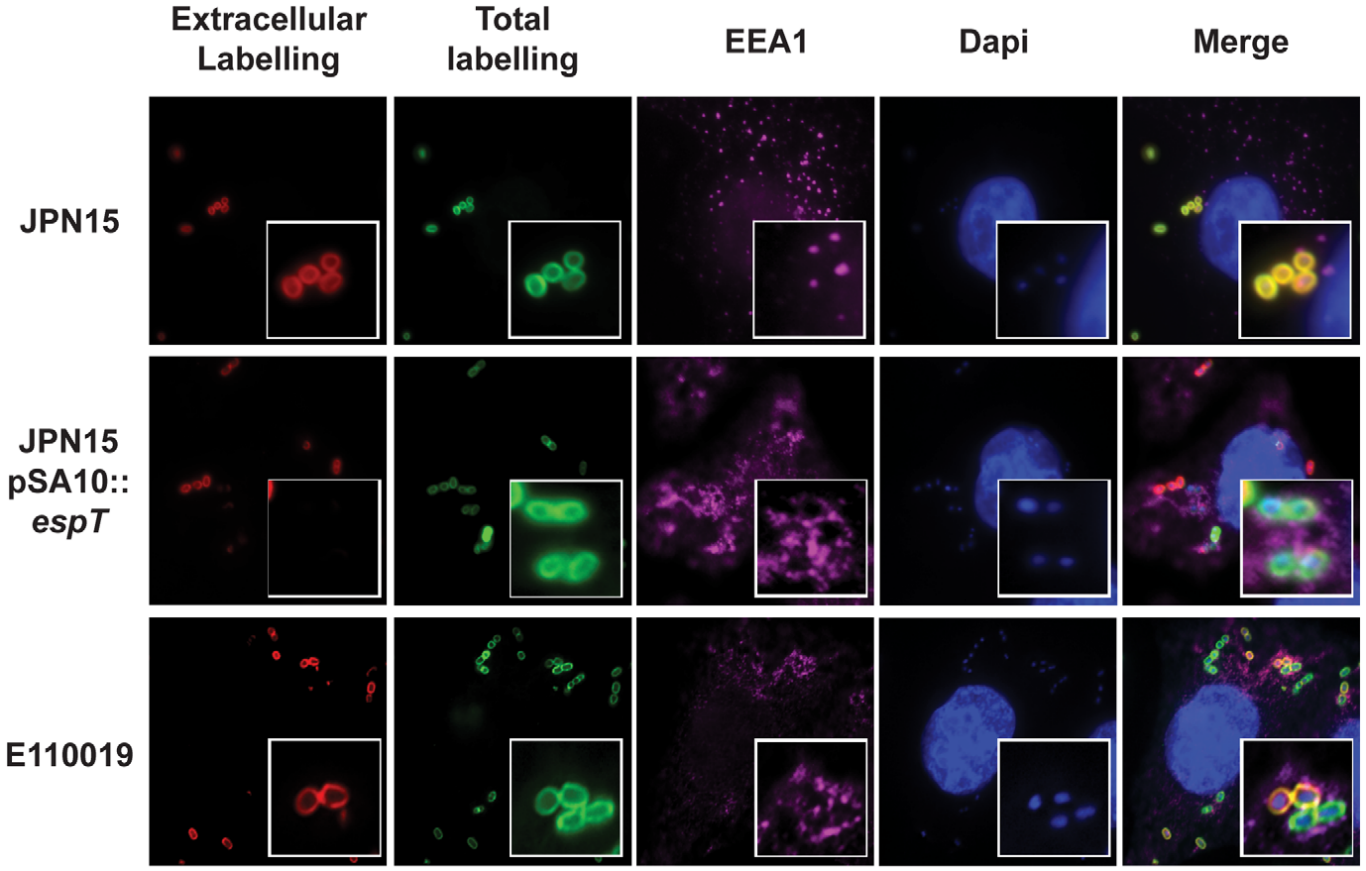
Internalized EPEC bacteria are enclosed within a vacuole. HeLa cells were infected with JPN15, JPN15 expressing EspT and E110019 for 45 min. Internalized bacteria bound within a vacuole were detected by anti EEA1 and a secondary antibody coupled to a CY5 (magenta). DNA was detected using Dapi staining. Extracellular JPN15 bacteria, which were detected by both the total stain and the extra-cellular probe, were not co-localized with EEA1. Intracellular JPN15 expressing EspT and E110019 which were detected by the total bacterial probe but not by the pre permeabilization stain were co localized within EEA1 rich structures indicative of early endosome like vacuoles.

**Figure 7:**
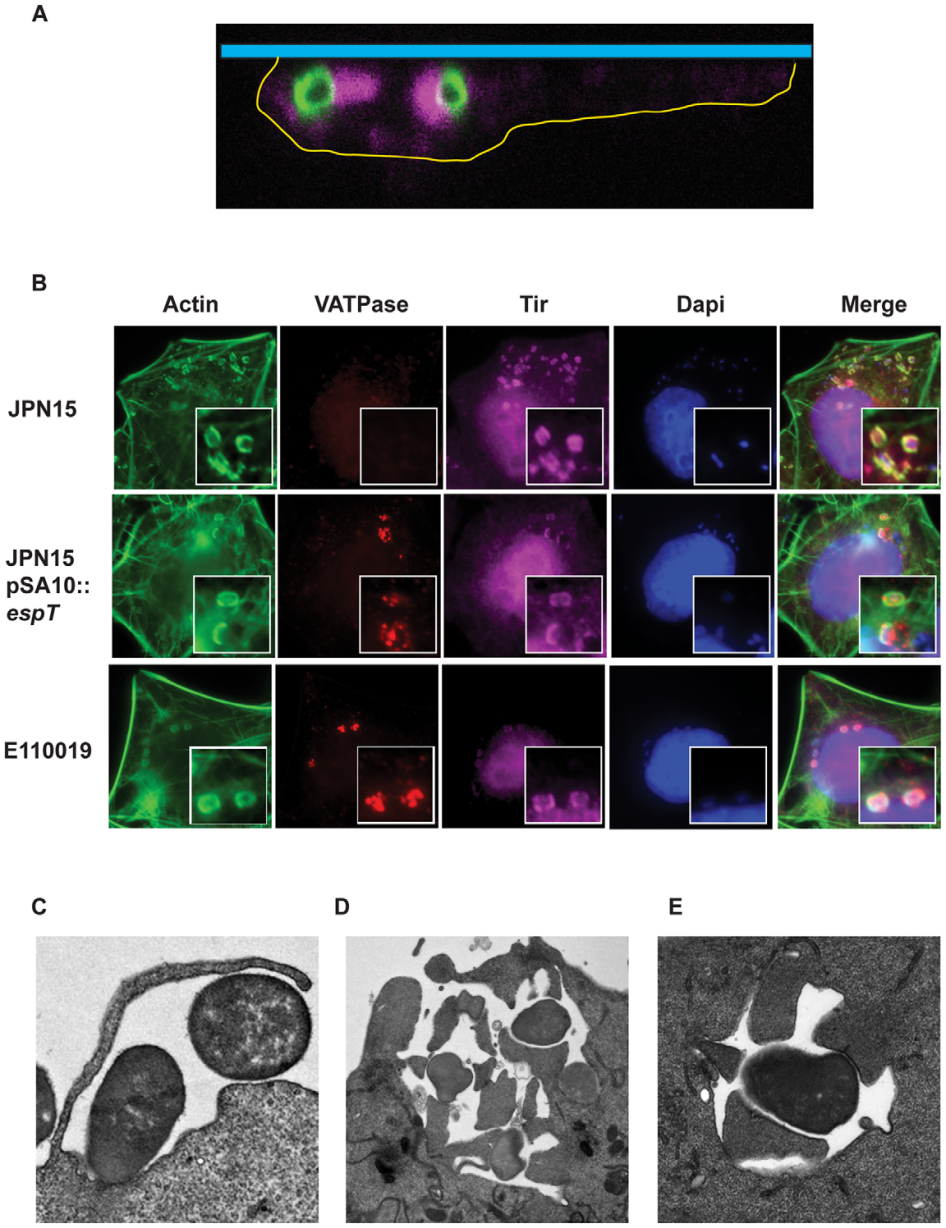
Internalized EPEC bacteria incorporate Tir into the vacuolar membrane which nucleates intracellular actin pedestals. (A) Swiss 3T3 cells were infected with E110019 and processed for immuno-fluorescence confocal microscopy. A series of confocal X-stacks were taken through the infected cells. The cell boundaries were defined based on actin staining and are marked by a yellow line, the coverslip is represented by the blue bar. The staining shows intracellular E110019 (Green) associated with actin pedestal-like structures (Magenta). (B) HeLa cells were infected for 60 min with JPN15, JPN15 expressing EspT, and E110019 were processed for immuno-fluorescence microscopy after 8 h gentamycin treatment. Actin was stained using Oregon Green phalloidin (Green), Tir was detected using polyclonal Tir antisera (Magenta), Vacuolar ATPase (VATPase) was detected using a monoclonal antibody (Red) and bacteria were detected using Dapi staining. Cells infected JPN15 recruited Tir to the site of bacterial attachment and form canonical actin rich pedestals but do not display any bacterial co-localization with the VATPase vacuolar marker. In cells infected with JPN15 expressing EspT or E110019 a proportion of bacteria were co-localized with VATPase, Tir and actin. (C) A TEM micrograph showing membrane ruffles engulfing E110019. (D) E110019 has the capacity to form multiple pedestals during ruffle formation and closure. (E) E110019 bacteria bound within a vacuole with intracellular pedestals formed around its circumference.

After the initial invasion of host cells internalized bacteria are often bound within a vacuole which resembles early endosomes (reviewed in [46]). Intracellular bacteria either remain within the vacuole or rapidly escape to the cytoplasm [26]. In order to determine whether invasive EPEC are bound within a vacuole or free in the cytoplasm HeLa cells were infected with JPN15, JPN15 over expressing EspT (pICC461) and E110019 for 5 min up to 24 h and stained with various vacuolar markers including Early Endosome Antigen 1 (EEA1), Vacuolar ATPase (VATPase) and Lamp1. Internalized JPN15 expressing EspT and E110019 were labeled with EEA1 while external bacteria and JPN15 lacking EspT were not (Fig. 6 shows staining at 45 min post infection). EEA1 staining was apparent after 5 min and persists up to 1 h post infection (data not shown). At 3 h and up to 12 h post infection the EPEC containing vacuole (ECV) was labeled with VATPase whilst external bacteria were not (Fig. 7B). Similarly to the *Salmonella* containing vacuole (SCV), a subset of ECVs became enriched with the lysosomal glycoprotein Lamp1 after 16 h (Fig. S7 and S8) and appear to adopt a perinuclear localization (Fig. 6B).

In order to determine if EPEC bacteria can multiply intracellularly we infected Swiss 3T3 cells with E110019 for 30 min before extracellular bacteria were killed by gentamycin. Infected cells were fixed for immuno-fluorescence microscopy at 2, 8, 16 and 24 h post infection. We observed a time dependent increase in the level of intracellular bacteria suggesting that internalized EPEC can multiply within host cells (Fig. S8).

### Internalized EPEC and *C. rodentium* form intracellular actin pedestals

After escaping from the vacuole many intracellular pathogens such as *Shigella*, *Burkholderia* and *Listeria* utilize specialized outer membrane proteins to recruit actin nucleating factors in order to produce a propulsive force (reviewed in [26]). EPEC is synonymous with actin nucleation which leads to formation of Tir-dependent actin rich pedestals [12]. During the course of this study we observed that invasive EPEC were associated with filamentous actin comets reminiscent of pedestals. Confocal X-stacks confirmed that the intracellular EPEC bacteria were associated with pedestal-like filamentous actin structures (Fig. 7A). In order to determine if Tir was localized at the actin nucleation sites, we infected HeLa cells for 1 h with JPN15, JPN15 expressing EspT and E110019; following washes the cells were treated with gentamycin for a further 8 h. The cells were then stained with anti-VATPase and anti-Tir antisera in conjunction with phalloidin and Dapi staining. HeLa cells infected with JPN15 exhibited extracellular, pedestal-associated, bacteria which were associated with Tir but not with VATPase (Fig. 7B). In contrast, internalized JPN15 expressing EspT and E110019 bacteria were co-labeled with anti-VATPase, actin and Tir (Fig. 7B). Similarly, invasive *C. rodentium* also formed intracellular pedestals (Fig. S5), while *C. rodentium* Δ*tir* was invasive but failed to trigger actin polymerization (Fig. S8). In addition, the intracellular EPEC Δ*escN*, internalized by ectopically expressing EspT, were not associated with actin pedestals (Fig. S6). These results suggest that the actin filaments associated with EPEC contained within the ECV is nucleated in a Tir-dependent mechanism analogous to pedestal formation by extracellular bacteria.

In order to confirm this assertion we infected HeLa cells with E110019 for 2 h and processed the cells for transmission electron microscopy (TEM). The TEM confirmed that E110019 bacteria are internalized via ruffle formation (Fig. 7C). E110019 can also be seen forming multiple pedestals with the membrane on opposing surfaces during ruffle formation and closure (Fig. 7D). Moreover, internalized EPEC bacteria contained within the ECV are associated actin pedestals, which are strikingly similar to those normally associated with extracellular EPEC (Fig. 7C–E). Interestingly, bacteria bound within ECVs can form multiple pedestals around their circumference (Fig. 7E).

In order to determine if the formation of intracellular pedestals by A/E pathogens plays a role in bacterial replication and survival within host cells we infected Swiss cells with wild type *C. rodentium* and *C. rodentium* Δ*tir* for 1.5 h and with E110019 for 30 min. Extracellular bacteria were then killed by a gentamycin wash and the cells incubated for a further 6, 12 or 24 h. We observed that both wild type *C. rodentium* and E110019 were capable of intracellular replication whereas the *C. rodentium* Δ*tir* mutant failed to replicate and instead exhibited a slow decline in bacterial numbers over time (Fig. S8). These results demonstrate that formation of pedestals by invasive A/E pathogens may play a functional role during intracellular survival.

## Discussion

A/E pathogens have been long considered to be extracellular bacteria which do not invade mammalian cells [47]. However, sporadic reports have shown that atypical EPEC strains can invade non-phagocytic cells [36],[48]. The invasive ability has been linked to intimin-Tir mediated tight association of EPEC with the host cell membrane which is hypothesized to produce immature phagocytosis cups leading to a passive push effect and inefficient internalization [35],[36]. In this study we demonstrated for the first time that EPEC can actively invade non-phagocytic cells by inducing formation of membrane ruffles, defining a new category of invasive EPEC. Furthermore we demonstrate that this phenomenon is dependent on the T3SS effector EspT which has previously shown activate Rac1 and Cdc42 [23]. We also show that both actin remodeling and invasion is dependent upon a functional EspT as JPN15 expressing a EspT^W63A^ failed to induce membrane ruffles or to invade. Importantly, in a previous report we indicated that expression of EspT might not confer bacterial invasion of epithelial cells [23]. However, these experiments were conducted using EPEC E2348/69, which in contrast to JPN15, *C. rodentium* and E110019, forms tight microclonies that mask the invasion phenotype (data not shown).

Intracellular pathogens have evolved a variety of mechanisms to promote invasion of mammalian cells, including the trigger (employed by *Salmonella* and *Shigella*) and zipper (employed by *Yersinia* and *Listeria*) mechanisms (reviewed in [26]). The trigger invasion mechanism is characterized by formation of actin rich membrane ruffles at the site of bacterial attachment, which are regulated by Rho GTPases, particularly Rac1 and Cdc42 and other cytoskeletal regulators such as PI3K [49]. *Shigella* and *Salmonella* utilize T3SS effector and translocator proteins such as IpgB1 and IpaC and SopB and SopE/2 to hijack host cell GTPase and phospho-inositol signaling to modulate membrane ruffling and formation of the macropinocytic pocket [28],[29],[30],[37]. Importantly, although both IpgB1 and EspT belong to the WxxxE family of effectors and play a prominent role in bacterial invasion by inducing membrane ruffles, we have recently shown that they activate Rac1 by distinct mechanisms [23].

Downstream of Rho GTPase signaling, membrane ruffle formation is nucleated by the WASP super family proteins including N-WASP and Wave2. *Salmonella* invasion has been demonstrated to be at least in part dependent upon the Arp2/3 binding activity of Wave2 and also the association of Wave2 with Abi1 [50]. Wave2 cannot bind Rac1 directly; two different mechanisms have been proposed to describe how a Wave2-Rac1 complex is formed. Innocenti *et al* and Steffen *et al* demonstrate that Wave2 binds to Abi1 and two accessory proteins PIR121 and Nap1 which mediate Rac1 binding [42],[51]. A report by Miki *et al* proposed that IRSp53 is the protein which links Rac1 to Wave2 [39]. In this study we demonstrate that EspT activation of Rac1 leads to a downstream recruitment of Wave2, Abi1 and IRSp53 to membrane ruffles. Depletion of endogenous Wave2 using siRNA resulted in a significant reduction in both the level of membrane ruffles induced by strains expressing EspT and their associated invasive capacity. We also show that the Arp2/3 and Abi1 binding regions of Wave2, but not N-WASP, are required for EspT-induced membrane ruffles and invasion. Furthermore, a construct of Wave2 which retained the IRSp53 and Arp2/3 binding regions but lacked the Abi1 interacting domain had a dominant negative effect on membrane ruffle formation, suggesting that IRSp53 is not required for, but may play an accessory role in, EspT-mediated actin rearrangements.

Once internalized *Shigella* and *Listeria* quickly escape the vacuole (reviewed in [52]). In contrast, *Salmonella* remains vacuole bound and utilizes different virulence factors to modify the vacuolar environment, position and interaction with the host endomembrane system in order to create an intracellular replicative SCV (reviewed in [53]). In this study we demonstrated that after invasion EPEC is bound within a vacuole (ECV) and remains vacuolated until at least 16 h post infection. We found that the ECV is EEA1 positive for up to 1 h post infection and progresses to being VATPase positive from 3 h to 12 h post infection. Furthermore, 12 h after infection the ECV appears to adopt a peri-nuclear position, which resembles the properties of the SCV. Similarly to the SCV (reviewed in [53]) we found that a subset of ECVs become enriched in the lysosomal glycoprotein Lamp1 (data not shown) indicating lysosomal fusion with the ECV. We also demonstrate that internalized EPEC bacteria can survive and replicate within host cells in a time dependent manner. Importantly and uniquely, we found that the ECV is associated with filamentous actin tails, which are reminiscent of the extracellular pedestals normally nucleated by EPEC stains. Formation of intracellular actin pedestal were essential for bacterial survival, as the intracellular population of invasive *tir* mutant declined over time.

Formation of extracellular pedestals is dependent upon the T3SS effector Tir [11]. The interaction of Tir with intimin triggers recruitment of the mammalian adaptor Nck which in turn recruits and activates N-WASP leading to Arp2/3 recruitment and actin polymerization [11],[54],[55],[56]. In this study we found that internalized EPEC can localize Tir to the vacuolar membrane in a T3SS dependent manner and that the localization of Tir can promote actin nucleation. Furthermore, we found that a *C. rodentium* Δ*tir* mutant is still invasive but does not form intracellular pedestals, demonstrating that pedestal formation by internalized bacteria is a Tir-dependent process analogous to that of extracellular bacteria. Additionally using TEM we found that invasive EPEC bound within a vacuole are associated with intracellular pedestals around the circumference of the bacteria. Interestingly, membrane ruffles seen engulfing invading EPEC were occasionally associated with pedestals, suggesting the pedestals can be formed during or after internalization.

Canonically actin is recruited to the surface of intracellular pathogens which are non-vacuolated and this recruitment is mediated by outer-membrane proteins which are free to interact with host cell signaling molecules present in the cytoplasm. For example following escape from the vacuole *Shigella* and *Listeria* utilize IcsA/VirG and ActA, respectively, to trigger actin polymerization and motility (reviewed in [57]). The Vaccinia virus uses the viral membrane protein A36R in order to generate actin based motility in a similar manner to the extracellular EPEC pedestals [58]. Importantly, the SCV is also associated with an actin nest which is required to maintain the integrity of the vacuole and support the intracellular replication of *Salmonella* (reviewed in [59]).

Due to the positioning of the actin extensions around the entire circumference of EPEC it is unlikely these intracellular pedestals are involved in classical actin-based motility. However, there are reports suggesting that actin polymerization and depolymerization around the periphery of E-cadherin-coated beads can lead to directional movement in process referred to as flashing [60]; for this reason at this stage we cannot rule out the possibility that intracellular pedestals confer actin based motility. Furthermore, formation of intracellular pedestals by invasive EPEC may play a role in maintaining the vacuole integrity in a similar way to that described for other vacuolated pathogens [59]. To the best of our knowledge the current study demonstrates for the first time that an intracellular bacteria is able to recruit filamentous actin comets to the pathogen cell surface whilst encapsulated in a vacuole.

In order to survive within intracellular niche vacuolated bacteria must evade host cell lysosome mediated degradation. Interestingly, during the course of this study we observed that internalized EPEC, which were enclosed in ECVs, displaying strong actin staining around their circumference were rarely Lamp1 positive, whereas ECVs which had little or no actin polymerization associated with them were homogenously labeled with Lamp1 (data not shown). Furthermore, we observed that a *C. rodentium* Δ*tir* mutant was attenuated for intracellular replication. We propose that formation of actin rich intracellular pedestals around the circumference of the ECV by invasive EPEC may constitute a physical barrier to lysosome fusion protecting the enclosed bacteria from lysosomal degradation; however this hypothesis requires further testing. A similar phenomenon has been described for the trafficking of endosomes and lysosomes to wounded sites of plasma membrane. At sites of plasma membrane disruption lysosomes and endosomes are recruited to seal the breach, this process is inhibited if the cortical actin meshwork is stabilized and enhanced when it is disrupted [61]. Similarly the lysosome dependent internalization of *Trypanosoma cruzi* requires a depolymerization of the cortical actin network to allow lysosome transit to the plasma membrane [62].

Recently, while screen ca. 1000 clinical EPEC and EHEC isolates we found that none of the EHEC strains and only 1.8% of the EPEC strains contain *espT* [21]. Interestingly, *espT* was found in EPEC E110019 which was linked to a particularly sever outbreak of gastroenteritis in Finland [22]. E110019 was found to be particularly infectious and unusually for EPEC was associated with person to person spread and adult disease [22]. Although we have no clinical data of the other *espT* positive isolates it is tempting to speculate that the expression of EspT could be at least in part responsible for the hyper virulence of the E110019 strain. Further studies of the invasive EPEC category are needed to assess the risk they pose to human health.

## Materials and Methods

### Bacteria strains

Bacterial strains used in this study are listed in Table 1. The *C. rodentium* Δ*espT* were constructed using the using the one-step PCR λ-red-mediated mutation protocol [63] The O111:H2 E110019 strain was isolated from an outbreak in Finland [22]. All the strains were maintained on Luria–Bertani (LB) broth or agar supplemented with ampicilin (100µg/ml) or Kanamycin (50 µg/ml).

**Table 1:** List of strains.

Strain	Description	Source/Reference
E110019	EPEC O111:H9 wild type	[22]
E2348/69	EPEC O127:H6 wild type	[66]
ICC192	EPEC O127:H6 Δ*escN*	[67]
JPN15	EPEC O127:H6 lacking the EAF plasmid encoding BFP	[38]
*Salmonella* Typhimurium	Strain SL1344	[68]
ICC169	*Citrobacter rodentium*	This study
ICC306	*Citrobacter rodentium ΔespT*	This study
ICC305	*Citrobacter rodentium Δtir*	R. Mundy

### Plasmids and molecular techniques

Plasmids used in this study are listed in Table 2; primers are listed in Table 3. *espT* was amplified by PCR using E110019 genomic DNA as template and cloned into pSA10 [64] (primer pair 1 and 2). All constructs were verified by DNA sequencing. Site directed mutagenesis of EspT was carried out using a Quickchange® II kit (Stratagene) and primers 3 and 4 according to the manufacturers instructions. Plasmids pSA10::*espT* was used as template for the mutagenic reactions. The pCX340 vector encoding EspT-TEM fusion was constructed after amplification of *espT* from *C. rodentium* using primer pair 5 and 6.

**Table 2:** List of plasmids.

Name	Description	Source/Reference
pSA10	pKK177-3 with Lac	[64]
pRK5::*myc-rac1N^17^*	Dominant negative Rac1	[69]
pRK5::*myc-cdc42N^17^*	Dominant negative Cdc42	[69]
pRK5:*myc-:wave2*	Wave2 full length	Gifted by Laura Machesky
pDSRED::*wave2ΔA*	Wave2 with truncated VCA	Gifted by Laura Machesky
pDSRED::*wave2ΔBP*	Wave2 with truncated WHD	Gifted by Laura Machesky
pICC228	pRK5::*espT-Myc*	[23]
pICC461	pSA10::*espT* E110019	This Study
pICC489	pACYC184::*espt C. rodentium*	This Study
pICC488	pCX340::*espT C. rodentium*	This Study
pICC490	pRK5::*espT* ^W63A^	This Study

**Table 3:** List of primers.

Primer	Name	Sequence
1	EspT-E110019-F	5′-ttgaattcatgccaggaacagtaaactcc-3′
2	EspT-E110019-R	5′-ccaatgcattggttctgcattaaacatattttaaatttctc-3′
3	EspTW63AF	5′-gaaaaacgaaggaaaaatgaatgaggcgatgagggaagaatgtatttgcttt-3′
4	EspTW63AR	5′-aaagcaaatacattcttccctcatcgcctcattcatttttccttcgtttttc-3′
5	EspTpCX340F	5′-ttcatatgccgggaacaataagctccag-3′
6	EspTpCX340R	5′-tgaattcggggttctctgcgcctcctgaa-3′

The mammalian expression vector pRK5 containing one of Rac^N17^ or Cdc42^N17^ dominant negatives used in the transfection assays were a gift from Nathalie Lamarche-Vane. The pRK5 encoding Wave2, and the pDSRED Wave2ΔA and Wave2ΔBP were a kind gift from Laura Machesky via Ray Carabeo.

### Infection of Swiss 3T3, HeLa and Caco2 cells

48 h prior to infection cells were seeded onto glass coverslips at a density of 5×10^5^ cells per well and maintained in DMEM supplemented with 10% FCS at 37°C in 5% CO_2_. Caco2 cells were grown in DMEM supplemented with 20% FCS at 37°C in 5% CO_2_. The cells were washed in PBS and the media changed every 24 h for 12 days until the cells polarized. 3 h before infection, the cells were washed 3 times with PBS, the media replaced with fresh DMEM without FCS supplemented with 1% mannose and 500 µl of primed bacteria were added to each well, the plates were then centrifuged at 1000 rpm for 5 min at room temperature and infections were carried out at 37°C in 5% CO_2_.

### β Lactamase (TEM) T3SS dependent translocation assay

HeLa cells were seeded on to glass coverslips for infection as previously described above. Wild type EPEC E2348/69 and Δ*escN* T3SS null mutant were transformed with the pCX340 vector encoding EspT-TEM-1 fusion; an NleD-TEM fusion was used as a positive control. Translocation assay was performed as described previously [65].

### Immunofluorescence staining and microscopy

Coverslips were washed 3 times in PBS and fixed with 3% Paraformaldehyde (PFA) for 15 min before washing 3 more times in PBS. For immuno-staining, the cells were permeabilized for 5 min in PBS 0.5% Triton X100, washed 3 times in PBS and quenched for 30 min with 50 mM NH_4_Cl. Pre prermeabilized samples were not treated with triton X100. The coverslips were then blocked for 1 h with PBS 0.5% BSA before incubation with primary and secondary antibodies. The primary antibody mouse anti EEA1 (BD biosciences) and mouse anti VATPase (Gifted by Prof. D. Holden) were used at a dilution of 1∶100, while rabbit anti O127, anti O111, anti O152, anti Tir and goat anti CSA-1 (salmonella LPS, gifted by Prof D. Holden) were used at a dilution of 1∶500. Rabbit anti Wave2 (SantaCruz Biotechnology) and Mouse anti Abi1 (Abcam) were used at 1∶200 dilutions. Coverslips were incubated with the primary antibody for 1 h, washed three times in PBS and incubated with the secondary antibodies. Donkey anti-rabbit IgG conjugated to a Cy2 or Cy3 fluorophore, donkey anti-mouse IgG conjugated to a Cy5 or Cy5 fluorophore, donkey anti goat IgG conjugated to a Cy2 or Cy3 fluorophore (Jackson laboratories) were used at a 1∶200. Actin was stained using AlexaFluor 633 phalloidin, Oregon Green phalloidin or Rhodamine phalliodin (Invitrogen) at a 1∶100 dilution. All dilutions were in PBS/0.5% BSA. Coverslips were mounted on slides using ProLong® Gold antifade reagent (Invitrogen) and visualized by Zeiss Axioimager immunofluorescence microscope using the following excitation wavelengths: Cy3 – 550nm, Cy5 – 650nm and Oregon Green – 488nm. All images were analyzed using the Axiovision Rel 4.5 software. Confocal X stacks were taken using a Leica Sp2 microscope. Cell boundaries were determined using actin staining and Abobe photoshop software.

### Transfection

Swiss 3T3 cells or HeLa cells were transfected with pRK5 encoding EspT, RhoA^N19^, Rac^N17^, Cdc42^N17^ dominant negatives fused to a Myc tag, pDSRED encoding Wave2, Wave2ΔA or Wave2ΔBP by lipofectamine 2000 (Invitrogen) according to the manufacturer's recommendations. The cells were incubated at 37°C in a humidified incubator with 5% CO_2_ for 16 h, washed twice in PBS before having their media replaced with DMEM as described previously. Transfected cells were infected with the appropriate strain as described above.

### siRNA of Wave2

HeLa cells were seeded at a density of approximatetly 5×10^6^ cells per well 24 h prior to transfection of either Wave2 siRNA pool or a non-targeting pool supplied by Dharmacon using HiPerFect (Qiagen) according to the manufacturers instructions. The media was changed 16 h after transfection and the cells were allowed to recover for 12 h before being trypsinated and seeded at a density of 5×10^6^ cells. The siRNA procedure was repeated for a total of 3 rounds before the cells were used. Levels of Wave2 and tubulin were then detected by western blotting using anti wave2 (Santa Cruz) and anti tubulin (Sigma) antibodies. Cells were then infected with the appropriate strain and processed for immuno-fluorescence microscopy as previously described.

### Gentamycin protection assay

Cells seeded into the wells of a 24 well plate were infected as described above for 6 h at 37°C in 5% CO_2_. The pre-gentamycin plates were washed 5 times in PBS and then permeabilzed for 15 minutes with 1% saponin in sterile water before plating in triplicate on LB plates in dilutions ranging from 10^0^ to 10^−7^. The post gentamycin samples were washed 5 times with PBS after the final wash the PBS was replaced with serum free DMEM containing 200µg/ml of gentmaycin and the cells incubated for 1 h at 37°C in 5% CO_2_. The plates were then washed a further 5 times in PBS before permeabilization and plating as described above. The pre and post gentamycin plates were then incubated for 15 h in a static 37°C incubator and the colony forming units (cfu) were counted. The percentage of invasion was calculated based on the ratio of cfu on the pre and post gentamycin plates.

### Scanning electron microscopy

Glass coverslips were seeded and infected for 2 h with the appropriate strains as described above. The cells were washed 3 times in phosphate buffer pH7.2 and then fixed with 2.5% Gluteraldehyde (Agar) in phosphate buffer pH7.2 for 15 min. The coverslips were then washed with phosphate buffer pH7.2 a further 3 times before being post fixed in 1% Osmium Tetroxide for 1 h. The cells were then washed 3 times in phosphate buffer before being washed for 15 min in graded ethanol solutions from 50% to 100% to dehydrate the samples. The cells were then transferred to an Emitech K850 Critical Point drier and processed according to the manufacturer's instructions. The coverslips were coated in gold/palladium mix using a Emitech Sc7620 minisputter to a thickness of approximately 370Å. Samples for scanning electron microscopy (SEM) were then examined blindly at an accelerating voltage of 25 kV using a Jeol JSM-6390.

### Transmission electron microscopy

6 well plates were seeded and infected for 2 h with the appropriate strains. The cells were washed 3 times in phosphate buffer pH7.2 and then fixed with 2.5% Gluteraldehyde in phosphate buffer pH7.2 for 15 min. The plates were then washed with phosphate buffer pH7.2 a further 3 times before being removed from the plate using a Teflon scraper and subsequently harvested into in eppendorf tube. The eppendorfs were then centrifuged at 10,000 RPM to pellet the cells. The cell pellets were post fixed in 1% Osmium Tetroxide for 1 h, followed by 1% buffered tannic acid for 30 min and then a 1% aqueous sodium sulfate rinse for 10 min. The sample was dehydrated using an ethanol-propylene oxide series (with 2% uranyl acetate added at the 30% step) and embedded in Epon-araldite for 24 h at 60°C. Ultrathin sections (60 nm) were cut with a Leica EMUC6 ultramicrotome, contrasted with uranyl acetate and lead citrate, and viewed with an FEI 120-kV Spirit Biotwin TEM. Images were obtained with a Tietz F415 digital TemCam.

## Supporting Information

Figure S1 EspT is translocated into host cells in a T3SS dependent manner. HeLa cells were infected with EPEC E2348/69 or E2348/69Δ*escN* containing the pCX340 β-lactamase fused to EspT. β-lactamase cleaves the CCF2/AM substrate, which fluoresces in green when uncleaved and in blue when cleaved, indicating translocation of the fusion protein. The T3SS effector NleD was used as a positive control.
(0.48 MB PDF)

Figure S2 IRSp53 is enriched in membrane ruffles induced by EspT. HeLa and Swiss cells were transfected with the ectopic expression vector pRK5 encoding EspT for 12 h. Actin was labeled with Oregon Green phalloidin (Green), Wave2 was detected with polyclonal rabbit antiserum (Red) and IRSp53 was detected using a monoclonal mouse antibody (Yellow). Transfection of EspT resulted in formation of membrane ruffles and lamellipodia in HeLa and Swiss cells respectively. Wave2 was localized to membrane ruffles and lamellipodia induced by EspT. IRSp53 was recruited to EspT dependent ruffles in HeLa cells but was not present in lamellipodia induced on Swiss 3T3 cells.
(1.52 MB PDF)

Figure S3 Wave2 WHD and VCA domains are needed for EspT-induced membrane remodeling. (A) Swiss cells were left untransfected or transfected with pDSRed encoding wild type Wave2 and Wave2ΔA (lacking the acidity Arp2/3 interacting region) or Wave2ΔBP (lacking the WHD needed for Abi1 binding). Transfected cells were infected with JPN15 expressing EspT for 2 h and processed for immuno-fluorescence microscopy. Actin was stained with Oregon green phalloidin (Green), the Wave constructs were detected with a polyclonal rabbit Wave2 antibody (Red) and JPN15 expressing EspT were visualized by Dapi. Mock transfected cells or cell transfected with wild type Wave2 displayed lamellipodia in 80–90% of transfected cells. Cells transfected with Wave2ΔA or Wave2ΔBP were severely attenuated in lamellipodia formation compared to the mock or Wave2 wild type transfected cells. (B) Quantification of lamellipodia and membrane ruffles on Swiss and HeLa cells respectively after 2 h infection with JPN15 expressing EspT. 100 cells were counted in triplicate in three independent experiments. Results are displayed as mean±SEM.
(2.59 MB PDF)

Figure S4 EspT mediated membrane remodeling and invasion is dependent on the conserved WxxxE motif. HeLa cells infected with JPN15, JPN15 expressing wild type EspT, or JPN15 expressing EspT^W63A^ for 3 h were fixed and stained with phalliodin (green) to detect actin and Dapi stain to label bacteria (blue). In cells infected with JPN15 and JPN15 expressing EspT^W63A^ there was no significant induction of membrane ruffling. Infection of HeLa cells with JPN15 expressing wild type EspT resulted in the formation of characteristic membrane ruffles. (B) Gentamycin protection assay of HeLa cells infected JPN15 and JPN15 expressing EspT or EspT^W63A^. Results are representative of 3 independent experiments carried out in duplicate and are displayed as mean±SEM.
(0.92 MB PDF)

Figure S5 EspT is an essential mediator of *C. rodentium* invasion of epithelial cells. HeLa cells infected with *C. rodentium*, *C. rodentium* Δ*espT* or complemented *C. rodentium* Δ*espT* were fixed and stained prior to permeabilization (extracellular labeling) (Red). The cells were then washed, permeabilized, re-labeled (Total labeling) (Green) along with Alexaflour 633 Phalloidin (Cyan) and Dapi (Blue). In cells infected with *C. rodentium* Δ*espT* all bacterial cells detected by the total stain were also labeled with the extracellular stain indicating that this strain was not invasive (highlighted with arrows). In cells infected with *C. rodentium* or *C. rodentium* Δ*espT* expressing EspT a significant proportion of bacteria labeled with the total probe were not strained with the extracellular probe demonstrating cells invasion (highlighted with arrows).
(1.73 MB PDF)

Figure S6 Ectopic expression of EspT can facilitate invasion of epithelial cells by a T3SS null mutant. HeLa cells were transfected with pRK5 encoding EspT and subsequently infected with a Δ*escN* T3SS mutant. The cells were then fixed and processed for immuno-fluorescence microscopy. Actin was stained using Alexafluor 633 phalloidin (Cyan), external and internal bacteria were labeled in red and green respectively. Ectopic expression of EspT led to the formation of actin rich membrane ruffles and a significant proportion of Δ*escN* bacteria became internalized (highlighted with arrows).
(0.90 MB PDF)

Figure S7 ECVs become Lamp1 positive at late time points of infection. HeLa cells were infected with E110019 for 30 min before the cells were washed with gentamycin to eliminate non invasive-bacteria. The infected cells were then incubated for a further 16 h. The cells were fixed and processed for immuno-fluorescence microscopy Lamp1 was detected with a monoclonal antibody (Cyan), actin was labelled with phalliodin (Red) and bacteria were detected with Dapi. There was accumulation of Lamp1 staining on ECVs at 16 h post infection, which was not apparent at earlier time points.
(0.75 MB PDF)

Figure S8 (A) Internalized EPEC survive and replicate in epithelial cells. HeLa cells were infected with E110019 for 30 min before the cells were washed with gentamycin to eliminate non invasive-bacteria. The cells were then incubated for 2, 8, 16 and 24 h in the presence of gentamycin. Cells were processed for immuno-fluorescence microscopy, bacteria were detected with Dapi (Dlue) and actin was labeled with phalliodin (Red). There was a time dependent increase in the level of intracellular bacteria. (B) Quantitative gentamycin protection assay of intracellular growth. HeLa cells were infected for 3 h with *C. rodentium*, *C. rodentium* Δ*tir* and E110019 before extracellualr bacteria were eliminated with gentamycin. Cells were then incubated in the presence of gentamycin for 6, 12 or 24 h before the cells were lysed and plated for CFU counting. Results are representative of 3 independent experiments and are presents as mean±SEM.
(1.92 MB PDF)

## References

[1] Nataro JP, Kaper JB (1998). Diarrheagenic Escherichia coli.. Clin Microbiol Rev.

[2] Mundy R, MacDonald TT, Dougan G, Frankel G, Wiles S (2005). Citrobacter rodentium of mice and man.. Cell Microbiol.

[3] Frankel G, Phillips AD (2008). Attaching effacing Escherichia coli and paradigms of Tir-triggered actin polymerization: getting off the pedestal.. Cell Microbiol.

[4] Knutton S, Lloyd DR, McNeish AS (1987). Adhesion of enteropathogenic Escherichia coli to human intestinal enterocytes and cultured human intestinal mucosa.. Infect Immun.

[5] Frankel G, Phillips AD, Rosenshine I, Dougan G, Kaper JB (1998). Enteropathogenic and enterohaemorrhagic Escherichia coli: more subversive elements.. Mol Microbiol.

[6] Garmendia J, Frankel G, Crepin VF (2005). Enteropathogenic and enterohemorrhagic Escherichia coli infections: translocation, translocation, translocation.. Infect Immun.

[7] Tobe T, Beatson SA, Taniguchi H, Abe H, Bailey CM (2006). An extensive repertoire of type III secretion effectors in Escherichia coli O157 and the role of lambdoid phages in their dissemination.. Proc Natl Acad Sci U S A.

[8] Iguchi A, Thomson NR, Ogura Y, Saunders D, Ooka T (2009). Complete genome sequence and comparative genome analysis of enteropathogenic Escherichia coli O127:H6 strain E2348/69.. J Bacteriol.

[9] Galan JE, Wolf-Watz H (2006). Protein delivery into eukaryotic cells by type III secretion machines.. Nature.

[10] Coburn B, Sekirov I, Finlay BB (2007). Type III secretion systems and disease.. Clin Microbiol Rev.

[11] Kenny B, DeVinney R, Stein M, Reinscheid DJ, Frey EA (1997). k.. Cell.

[12] Frankel G, Phillips AD, Trabulsi LR, Knutton S, Dougan G (2001). Intimin and the host cell–is it bound to end in Tir(s)?. Trends Microbiol.

[13] Matsuzawa T, Kuwae A, Yoshida S, Sasakawa C, Abe A (2004). Enteropathogenic Escherichia coli activates the RhoA signaling pathway via the stimulation of GEF-H1.. Embo J.

[14] Kenny B, Jepson M (2000). Targeting of an enteropathogenic Escherichia coli (EPEC) effector protein to host mitochondria.. Cell Microbiol.

[15] Arbeloa A, Bulgin RR, MacKenzie G, Shaw RK, Pallen MJ (2008). Subversion of actin dynamics by EspM effectors of attaching and effacing bacterial pathogens.. Cell Microbiol.

[16] Alto NM, Shao F, Lazar CS, Brost RL, Chua G (2006). Identification of a bacterial type III effector family with G protein mimicry functions.. Cell.

[17] Berger CN, Crepin VF, Jepson MA, Arbeloa A, Frankel G (2009). The mechanisms used by enteropathogenic Escherichia coli to control filopodia dynamics.. Cell Microbiol.

[18] Jaffe AB, Hall A (2002). Rho GTPases in transformation and metastasis.. Adv Cancer Res.

[19] Etienne-Manneville S, Hall A (2002). Rho GTPases in cell biology.. Nature.

[20] Jaffe AB, Hall A (2005). Rho GTPases: biochemistry and biology.. Annu Rev Cell Dev Biol.

[21] Arbeloa A, Blanco M, Moreira FC, Bulgin R, Lopez C (2009). Distribution of espM and espT among enteropathogenic and enterohaemorrhagic Escherichia coli.. J Med Microbiol.

[22] Viljanen MK, Peltola T, Junnila SY, Olkkonen L, Jarvinen H (1990). Outbreak of diarrhoea due to Escherichia coli O111:B4 in schoolchildren and adults: association of Vi antigen-like reactivity.. Lancet.

[23] Bulgin RR, Arbeloa A, Chung JC, Frankel G (2009). EspT triggers formation of lamellipodia and membrane ruffles through activation of Rac-1 and Cdc42.. Cell Microbiol.

[24] Buccione R, Orth JD, McNiven MA (2004). Foot and mouth: podosomes, invadopodia and circular dorsal ruffles.. Nat Rev Mol Cell Biol.

[25] Chhabra ES, Higgs HN (2007). The many faces of actin: matching assembly factors with cellular structures.. Nat Cell Biol.

[26] Cossart P, Sansonetti PJ (2004). Bacterial invasion: the paradigms of enteroinvasive pathogens.. Science.

[27] Yoshida S, Sasakawa C (2003). Exploiting host microtubule dynamics: a new aspect of bacterial invasion.. Trends Microbiol.

[28] Hardt WD, Chen LM, Schuebel KE, Bustelo XR, Galan JE (1998). S. typhimurium encodes an activator of Rho GTPases that induces membrane ruffling and nuclear responses in host cells.. Cell.

[29] Patel JC, Galan JE (2006). Differential activation and function of Rho GTPases during Salmonella-host cell interactions.. J Cell Biol.

[30] Tran Van Nhieu G, Caron E, Hall A, Sansonetti PJ (1999). IpaC induces actin polymerization and filopodia formation during Shigella entry into epithelial cells.. Embo J.

[31] Mounier J, Popoff MR, Enninga J, Frame MC, Sansonetti PJ (2009). The IpaC carboxyterminal effector domain mediates Src-dependent actin polymerization during Shigella invasion of epithelial cells.. PLoS Pathog.

[32] Burton EA, Plattner R, Pendergast AM (2003). Abl tyrosine kinases are required for infection by Shigella flexneri.. Embo J.

[33] Handa Y, Suzuki M, Ohya K, Iwai H, Ishijima N (2007). Shigella IpgB1 promotes bacterial entry through the ELMO-Dock180 machinery.. Nat Cell Biol.

[34] Drucker MM, Polliack A, Yeivin R, Sacks TG (1970). Immunofluorescent demonstration of enteropathogenic Escherichia coli in tissues of infants dying with enteritis.. Pediatrics.

[35] Donnenberg MS, Kaper JB (1992). Enteropathogenic Escherichia coli.. Infect Immun.

[36] Hernandes RT, Silva RM, Carneiro SM, Salvador FA, Fernandes MC (2008). The localized adherence pattern of an atypical enteropathogenic Escherichia coli is mediated by intimin omicron and unexpectedly promotes HeLa cell invasion.. Cell Microbiol.

[37] Ohya K, Handa Y, Ogawa M, Suzuki M, Sasakawa C (2005). IpgB1 is a novel Shigella effector protein involved in bacterial invasion of host cells. Its activity to promote membrane ruffling via Rac1 and Cdc42 activation.. J Biol Chem.

[38] Jerse AE, Yu J, Tall BD, Kaper JB (1990). A genetic locus of enteropathogenic Escherichia coli necessary for the production of attaching and effacing lesions on tissue culture cells.. Proc Natl Acad Sci U S A.

[39] Miki H, Yamaguchi H, Suetsugu S, Takenawa T (2000). IRSp53 is an essential intermediate between Rac and WAVE in the regulation of membrane ruffling.. Nature.

[40] Legg JA, Bompard G, Dawson J, Morris HL, Andrew N (2007). N-WASP involvement in dorsal ruffle formation in mouse embryonic fibroblasts.. Mol Biol Cell.

[41] Machuy N, Campa F, Thieck O, Rudel T (2007). c-Abl-binding protein interacts with p21-activated kinase 2 (PAK-2) to regulate PDGF-induced membrane ruffles.. J Mol Biol.

[42] Innocenti M, Zucconi A, Disanza A, Frittoli E, Areces LB (2004). Abi1 is essential for the formation and activation of a WAVE2 signalling complex.. Nat Cell Biol.

[43] Suetsugu S, Kurisu S, Oikawa T, Yamazaki D, Oda A (2006). Optimization of WAVE2 complex-induced actin polymerization by membrane-bound IRSp53, PIP(3), and Rac.. J Cell Biol.

[44] Suetsugu S, Miki H, Takenawa T (1999). Identification of two human WAVE/SCAR homologues as general actin regulatory molecules which associate with the Arp2/3 complex.. Biochem Biophys Res Commun.

[45] Takenawa T, Miki H (2001). WASP and WAVE family proteins: key molecules for rapid rearrangement of cortical actin filaments and cell movement.. J Cell Sci.

[46] Kumar Y, Valdivia RH (2009). Leading a sheltered life: intracellular pathogens and maintenance of vacuolar compartments.. Cell Host Microbe.

[47] Celli J, Deng W, Finlay BB (2000). Enteropathogenic Escherichia coli (EPEC) attachment to epithelial cells: exploiting the host cell cytoskeleton from the outside.. Cell Microbiol.

[48] Donnenberg MS, Donohue-Rolfe A, Keusch GT (1989). Epithelial cell invasion: an overlooked property of enteropathogenic Escherichia coli (EPEC) associated with the EPEC adherence factor.. J Infect Dis.

[49] Ladwein M, Rottner K (2008). On the Rho'd: the regulation of membrane protrusions by Rho-GTPases.. FEBS Lett.

[50] Shi J, Scita G, Casanova JE (2005). WAVE2 signaling mediates invasion of polarized epithelial cells by Salmonella typhimurium.. J Biol Chem.

[51] Steffen A, Rottner K, Ehinger J, Innocenti M, Scita G (2004). Sra-1 and Nap1 link Rac to actin assembly driving lamellipodia formation.. Embo J.

[52] Schroeder GN, Hilbi H (2008). Molecular pathogenesis of Shigella spp.: controlling host cell signaling, invasion, and death by type III secretion.. Clin Microbiol Rev.

[53] Steele-Mortimer O (2008). The Salmonella-containing vacuole: moving with the times.. Curr Opin Microbiol.

[54] Campellone KG, Giese A, Tipper DJ, Leong JM (2002). A tyrosine-phosphorylated 12-amino-acid sequence of enteropathogenic Escherichia coli Tir binds the host adaptor protein Nck and is required for Nck localization to actin pedestals.. Mol Microbiol.

[55] Gruenheid S, DeVinney R, Bladt F, Goosney D, Gelkop S (2001). Enteropathogenic E. coli Tir binds Nck to initiate actin pedestal formation in host cells.. Nat Cell Biol.

[56] Lommel S, Benesch S, Rottner K, Franz T, Wehland J (2001). Actin pedestal formation by enteropathogenic Escherichia coli and intracellular motility of Shigella flexneri are abolished in N-WASP-defective cells.. EMBO Rep.

[57] Cossart P (1995). Actin-based bacterial motility.. Curr Opin Cell Biol.

[58] Frischknecht F, Moreau V, Rottger S, Gonfloni S, Reckmann I (1999). Actin-based motility of vaccinia virus mimics receptor tyrosine kinase signalling.. Nature.

[59] Guiney DG, Lesnick M (2005). Targeting of the actin cytoskeleton during infection by Salmonella strains.. Clin Immunol.

[60] Yam PT, Theriot JA (2004). Repeated cycles of rapid actin assembly and disassembly on epithelial cell phagosomes.. Mol Biol Cell.

[61] Miyake K, McNeil PL, Suzuki K, Tsunoda R, Sugai N (2001). An actin barrier to resealing.. J Cell Sci.

[62] Rodriguez A, Rioult MG, Ora A, Andrews NW (1995). A trypanosome-soluble factor induces IP3 formation, intracellular Ca2+ mobilization and microfilament rearrangement in host cells.. J Cell Biol.

[63] Datsenko KA, Wanner BL (2000). One-step inactivation of chromosomal genes in Escherichia coli K-12 using PCR products.. Proc Natl Acad Sci U S A.

[64] Schlosser-Silverman E, Elgrably-Weiss M, Rosenshine I, Kohen R, Altuvia S (2000). Characterization of Escherichia coli DNA lesions generated within J774 macrophages.. J Bacteriol.

[65] Charpentier X, Oswald E (2004). Identification of the secretion and translocation domain of the enteropathogenic and enterohemorrhagic Escherichia coli effector Cif, using TEM-1 beta-lactamase as a new fluorescence-based reporter.. J Bacteriol.

[66] Levine MM, Bergquist EJ, Nalin DR, Waterman DH, Hornick RB (1978). Escherichia coli strains that cause diarrhoea but do not produce heat-labile or heat-stable enterotoxins and are non-invasive.. Lancet.

[67] Garmendia J, Phillips AD, Carlier MF, Chong Y, Schuller S (2004). TccP is an enterohaemorrhagic Escherichia coli O157:H7 type III effector protein that couples Tir to the actin-cytoskeleton.. Cell Microbiol.

[68] Hueck CJ, Hantman MJ, Bajaj V, Johnston C, Lee CA (1995). Salmonella typhimurium secreted invasion determinants are homologous to Shigella Ipa proteins.. Mol Microbiol.

[69] Pelletier S, Julien C, Popoff MR, Lamarche-Vane N, Meloche S (2005). Cyclic AMP induces morphological changes of vascular smooth muscle cells by inhibiting a Rac-dependent signaling pathway.. J Cell Physiol.

