# Supplementary figures and images for: The T3SS Effector EspT Defines a New Category of Invasive Enteropathogenic E. coli (EPEC) Which Form Intracellular Actin Pedestals
Source: PLoS Pathog. 2009 Dec 11;5(12):e1000683. doi: 10.1371/journal.ppat.1000683 (PMC2782363; doi:10.1371/journal.ppat.1000683)

S1

EPEC pCX340::*NleD*

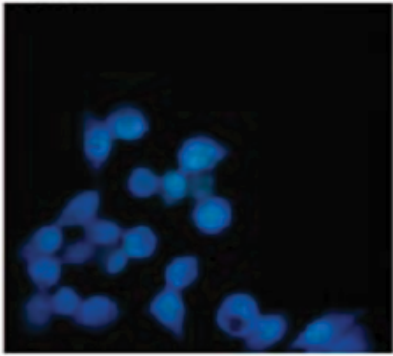

EPECDescN pCX340::*espT*

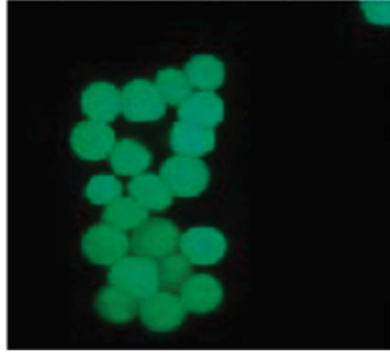

EPEC pCX340::*espT*

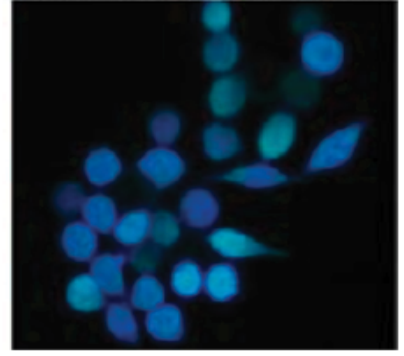

Supplement: Figure S1 — EspT is translocated into host cells in a T3SS dependent manner. HeLa cells were infected with EPEC E2348/69 or E2348/69ΔescN containing the pCX340 β-lactamase fused to EspT. β-lactamase cleaves the CCF2/AM substrate, which fluoresces in green when uncleaved and in blue when cleaved, indicating translocation of the fusion protein. The T3SS effector NleD was used as a positive control. (0.48 MB PDF) [file ppat.1000683.s001.pdf]

**S2**

**Actin**

**Wave2**

**IRSp53**

**Merge**

**pRK5::  
*espT*  
HeLa**

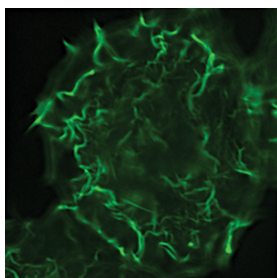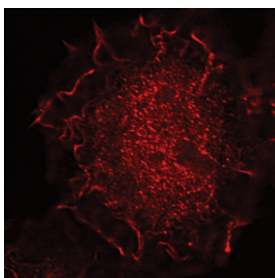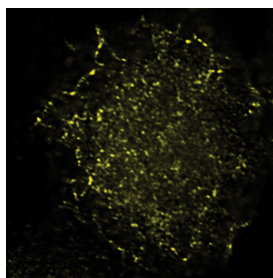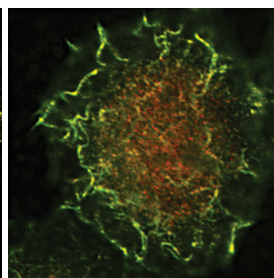

**pRK5::  
*espT*  
Swiss 3T3**

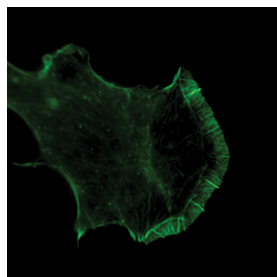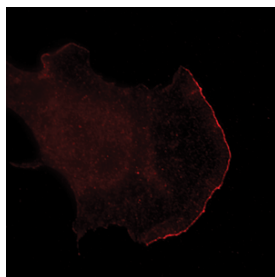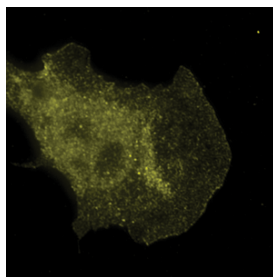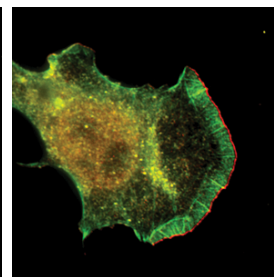

Supplement: Figure S2 — IRSp53 is enriched in membrane ruffles induced by EspT. HeLa and Swiss cells were transfected with the ectopic expression vector pRK5 encoding EspT for 12 h. Actin was labeled with Oregon Green phalloidin (Green), Wave2 was detected with polyclonal rabbit antiserum (Red) and IRSp53 was detected using a monoclonal mouse antibody (Yellow). Transfection of EspT resulted in formation of membrane ruffles and lamellipodia in HeLa and Swiss cells respectively. Wave2 was localized to membrane ruffles and lamellipodia induced by EspT. IRSp53 was recruited to EspT dependent ruffles in HeLa cells but was not present in lamellipodia induced on Swiss 3T3 cells. (1.52 MB PDF) [file ppat.1000683.s002.pdf]

**S3**

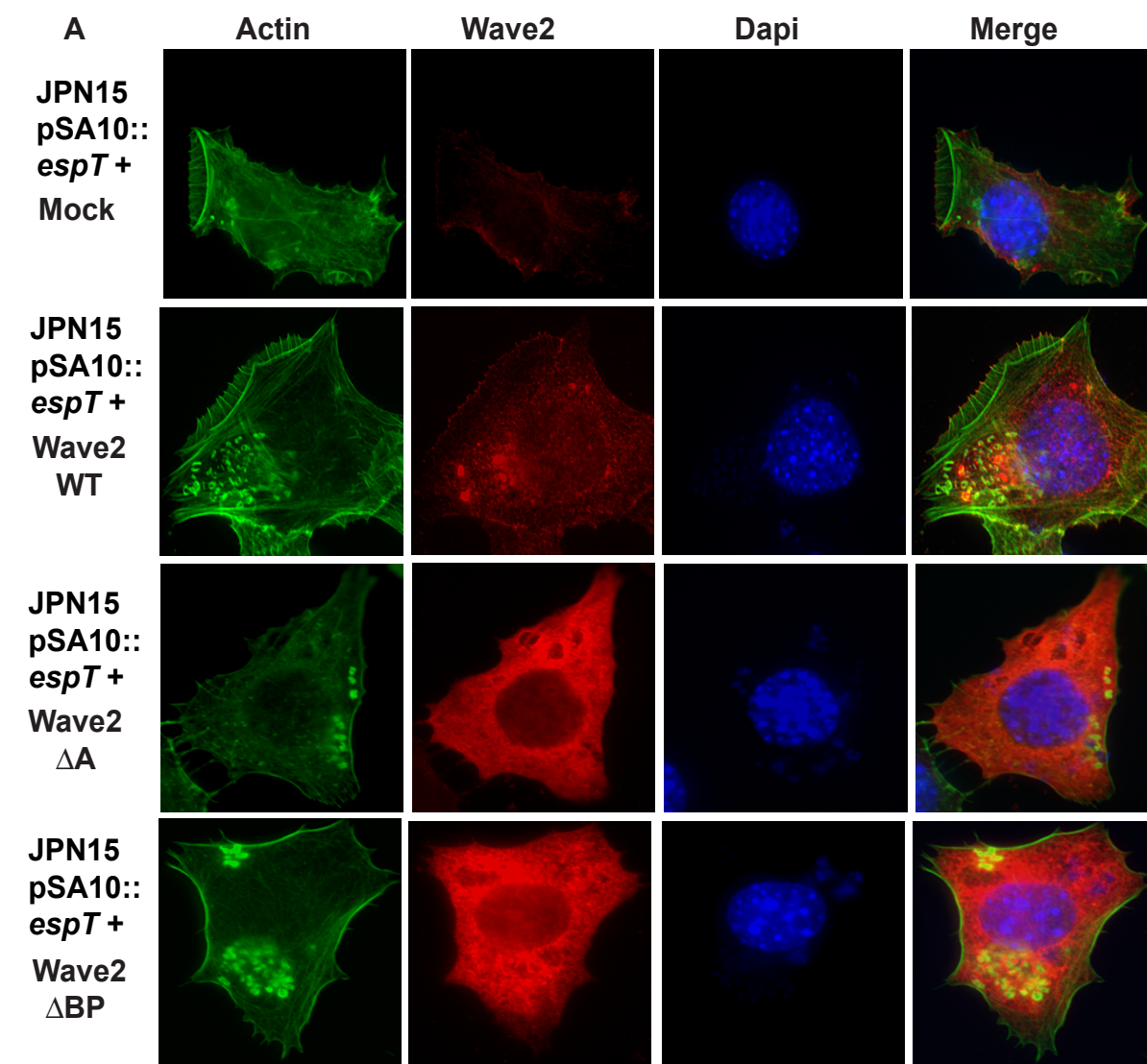

**B**

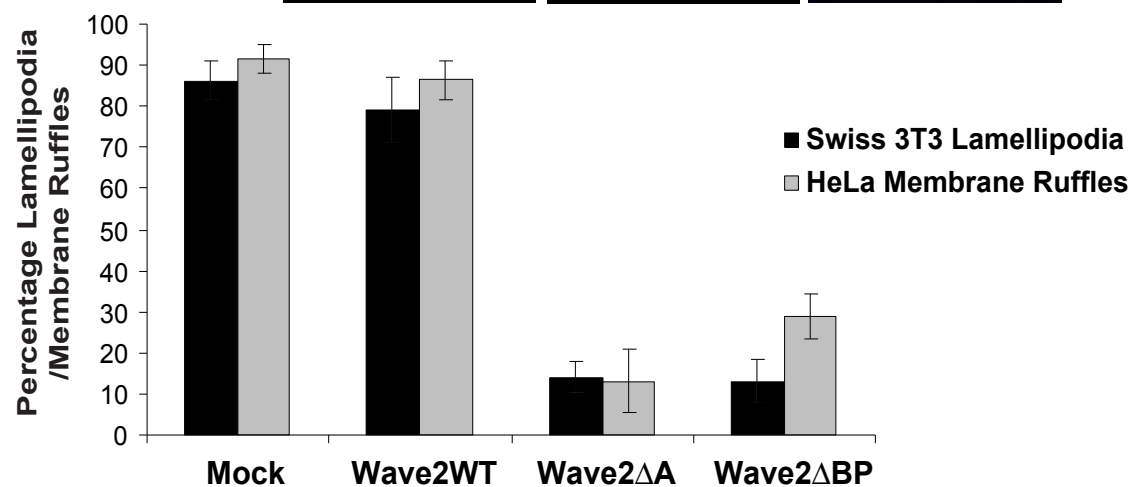

Supplement: Figure S3 — Wave2 WHD and VCA domains are needed for EspT-induced membrane remodeling. (A) Swiss cells were left untransfected or transfected with pDSRed encoding wild type Wave2 and Wave2ΔA (lacking the acidity Arp2/3 interacting region) or Wave2ΔBP (lacking the WHD needed for Abi1 binding). Transfected cells were infected with JPN15 expressing EspT for 2 h and processed for immuno-fluorescence microscopy. Actin was stained with Oregon green phalloidin (Green), the Wave constructs were detected with a polyclonal rabbit Wave2 antibody (Red) and JPN15 expressing EspT were visualized by Dapi. Mock transfected cells or cell transfected with wild type Wave2 displayed lamellipodia in 80–90% of transfected cells. Cells transfected with Wave2ΔA or Wave2ΔBP were severely attenuated in lamellipodia formation compared to the mock or Wave2 wild type transfected cells. (B) Quantification of lamellipodia and membrane ruffles on Swiss and HeLa cells respectively after 2 h infection with JPN15 expressing EspT. 100 cells were counted in triplicate in three independent experiments. Results are displayed as mean±SEM. (2.59 MB PDF) [file ppat.1000683.s003.pdf]

S4

A

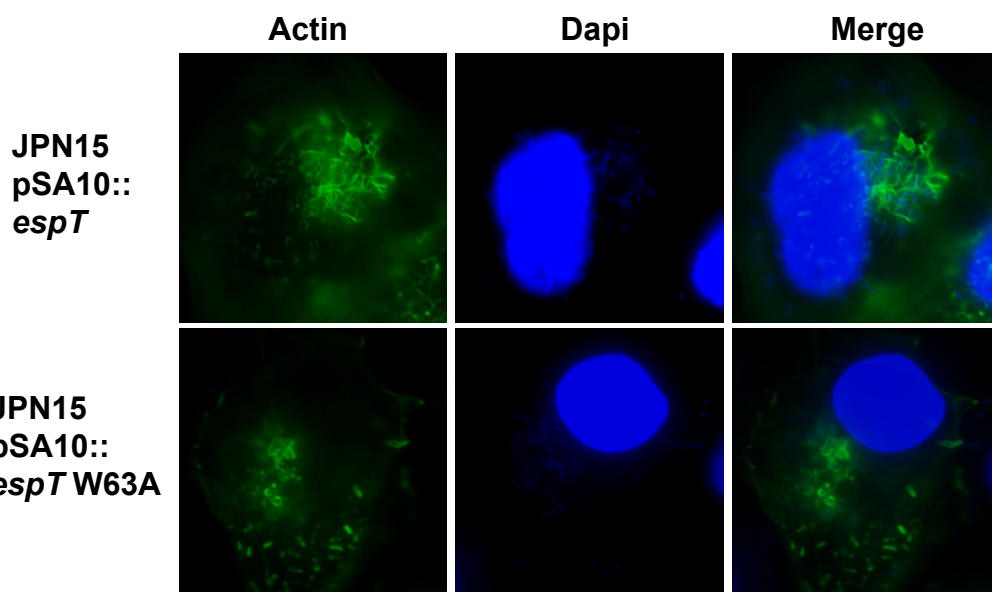

B

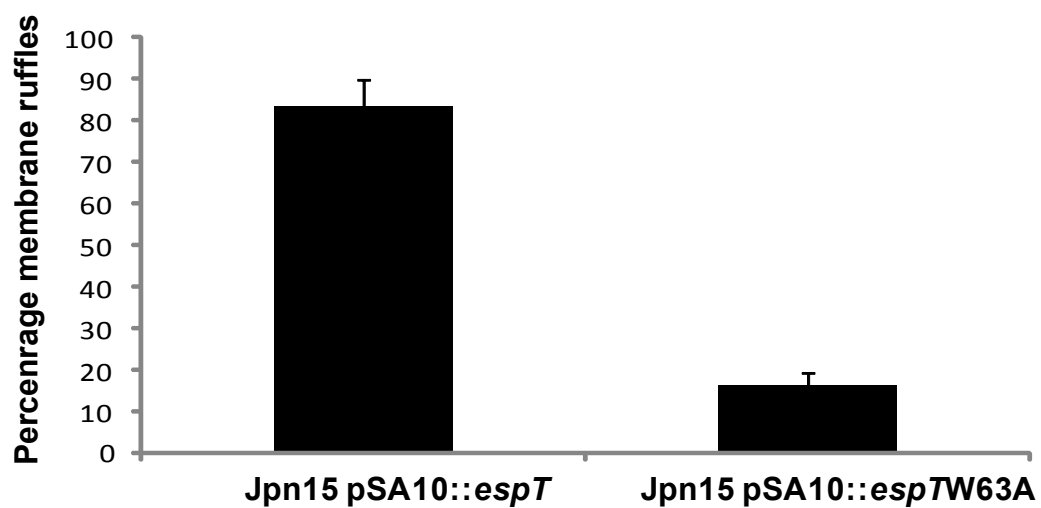

C

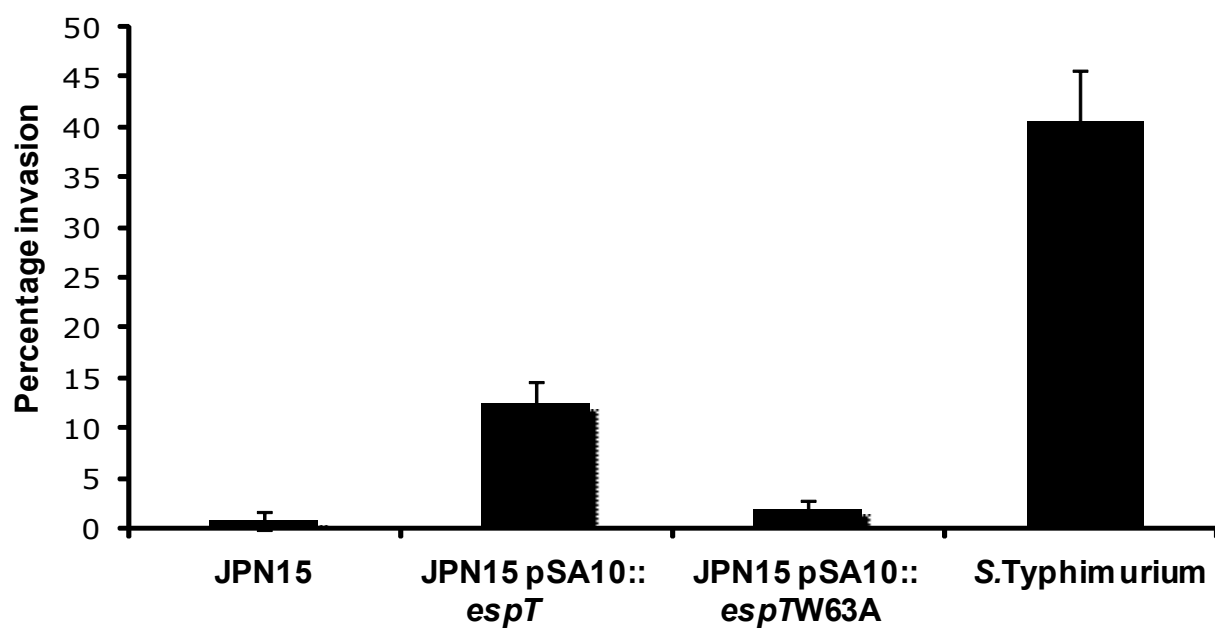

Supplement: Figure S4 — EspT mediated membrane remodeling and invasion is dependent on the conserved WxxxE motif. HeLa cells infected with JPN15, JPN15 expressing wild type EspT, or JPN15 expressing EspTW63A for 3 h were fixed and stained with phalliodin (green) to detect actin and Dapi stain to label bacteria (blue). In cells infected with JPN15 and JPN15 expressing EspTW63A there was no significant induction of membrane ruffling. Infection of HeLa cells with JPN15 expressing wild type EspT resulted in the formation of characteristic membrane ruffles. (B) Gentamycin protection assay of HeLa cells infected JPN15 and JPN15 expressing EspT or EspTW63A. Results are representative of 3 independent experiments carried out in duplicate and are displayed as mean±SEM. (0.92 MB PDF) [file ppat.1000683.s004.pdf]

S5

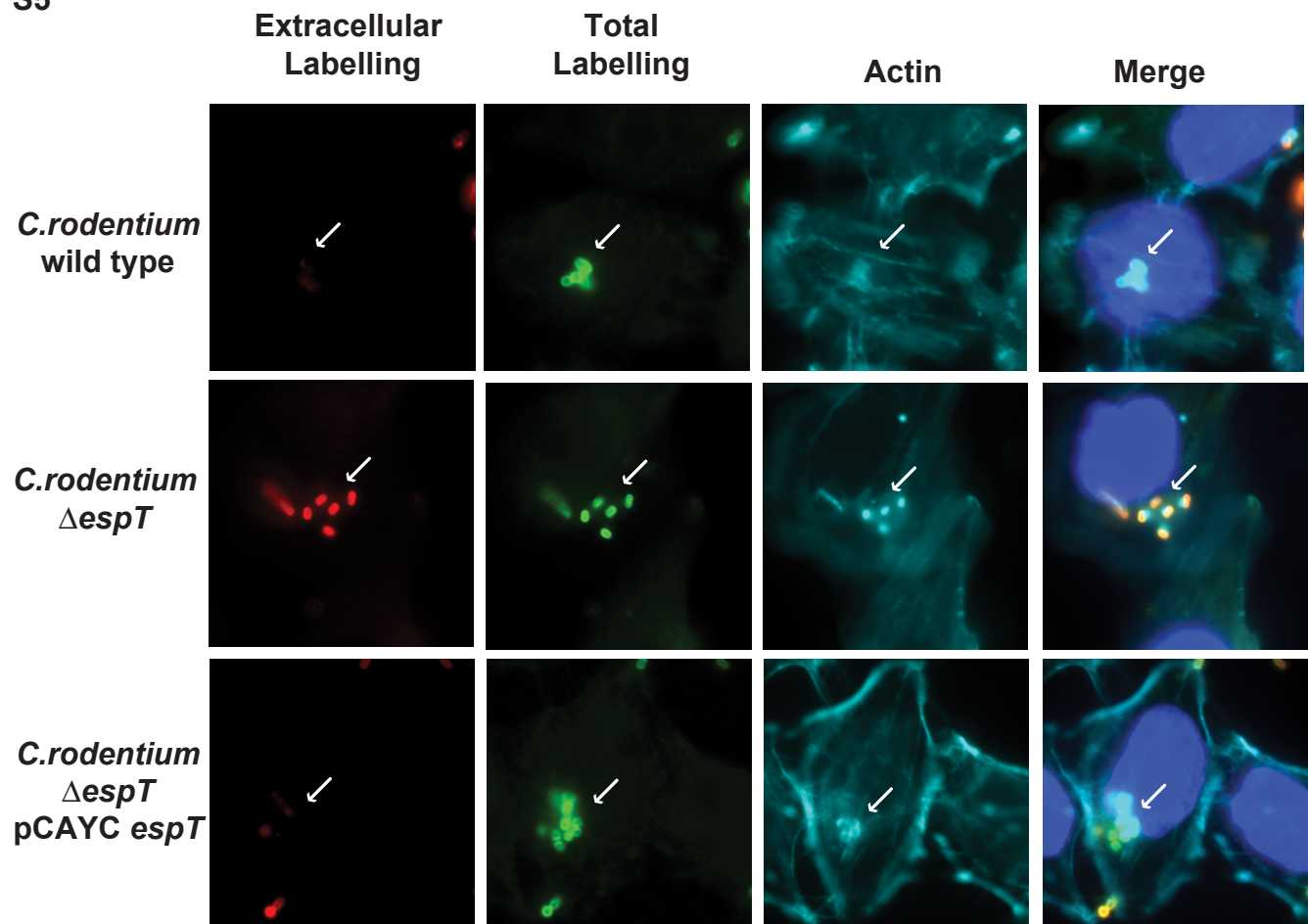

Supplement: Figure S5 — EspT is an essential mediator of C. rodentium invasion of epithelial cells. HeLa cells infected with C. rodentium, C. rodentium ΔespT or complemented C. rodentium ΔespT were fixed and stained prior to permeabilization (extracellular labeling) (Red). The cells were then washed, permeabilized, re-labeled (Total labeling) (Green) along with Alexaflour 633 Phalloidin (Cyan) and Dapi (Blue). In cells infected with C. rodentium ΔespT all bacterial cells detected by the total stain were also labeled with the extracellular stain indicating that this strain was not invasive (highlighted with arrows). In cells infected with C. rodentium or C. rodentium ΔespT expressing EspT a significant proportion of bacteria labeled with the total probe were not strained with the extracellular probe demonstrating cells invasion (highlighted with arrows). (1.73 MB PDF) [file ppat.1000683.s005.pdf]

S6

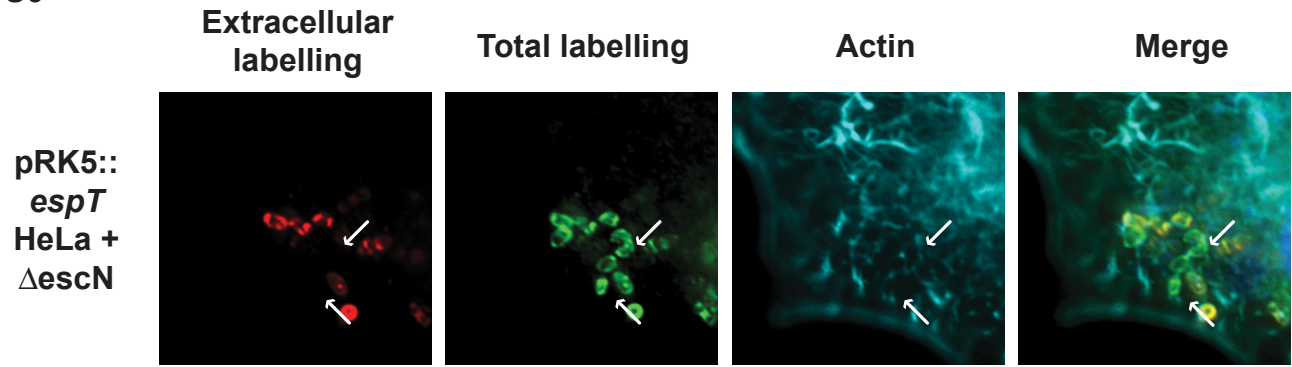

Supplement: Figure S6 — Ectopic expression of EspT can facilitate invasion of epithelial cells by a T3SS null mutant. HeLa cells were transfected with pRK5 encoding EspT and subsequently infected with a ΔescN T3SS mutant. The cells were then fixed and processed for immuno-fluorescence microscopy. Actin was stained using Alexafluor 633 phalloidin (Cyan), external and internal bacteria were labeled in red and green respectively. Ectopic expression of EspT led to the formation of actin rich membrane ruffles and a significant proportion of ΔescN bacteria became internalized (highlighted with arrows). (0.90 MB PDF) [file ppat.1000683.s006.pdf]

**S7**

**Actin**

**Lamp1**

**Dapi**

**Merge**

**E110019  
16 hours**

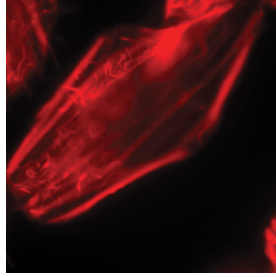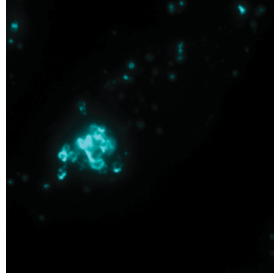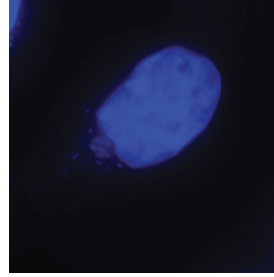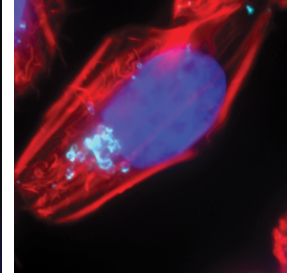

Supplement: Figure S7 — ECVs become Lamp1 positive at late time points of infection. HeLa cells were infected with E110019 for 30 min before the cells were washed with gentamycin to eliminate non invasive-bacteria. The infected cells were then incubated for a further 16 h. The cells were fixed and processed for immuno-fluorescence microscopy Lamp1 was detected with a monoclonal antibody (Cyan), actin was labelled with phalliodin (Red) and bacteria were detected with Dapi. There was accumulation of Lamp1 staining on ECVs at 16 h post infection, which was not apparent at earlier time points. (0.75 MB PDF) [file ppat.1000683.s007.pdf]

S 8 A

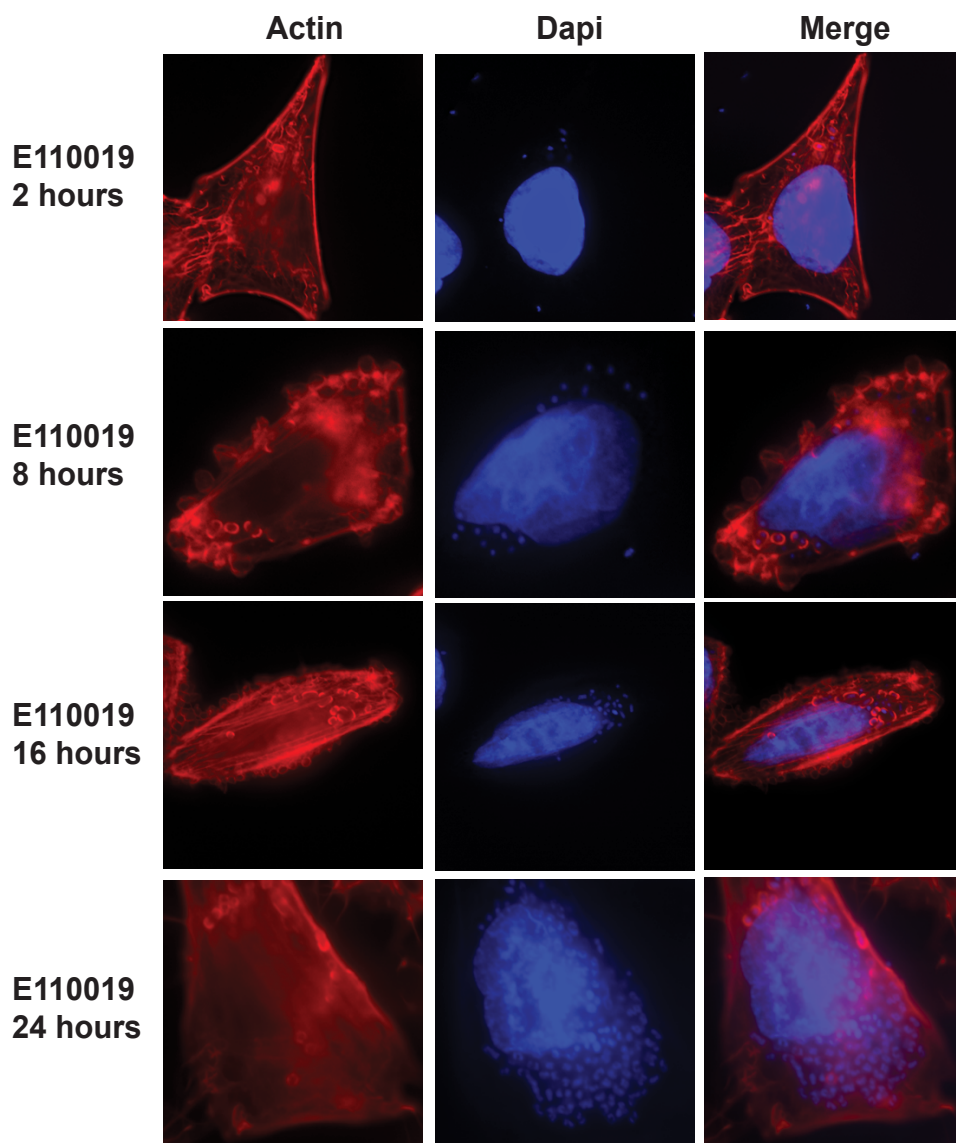

B

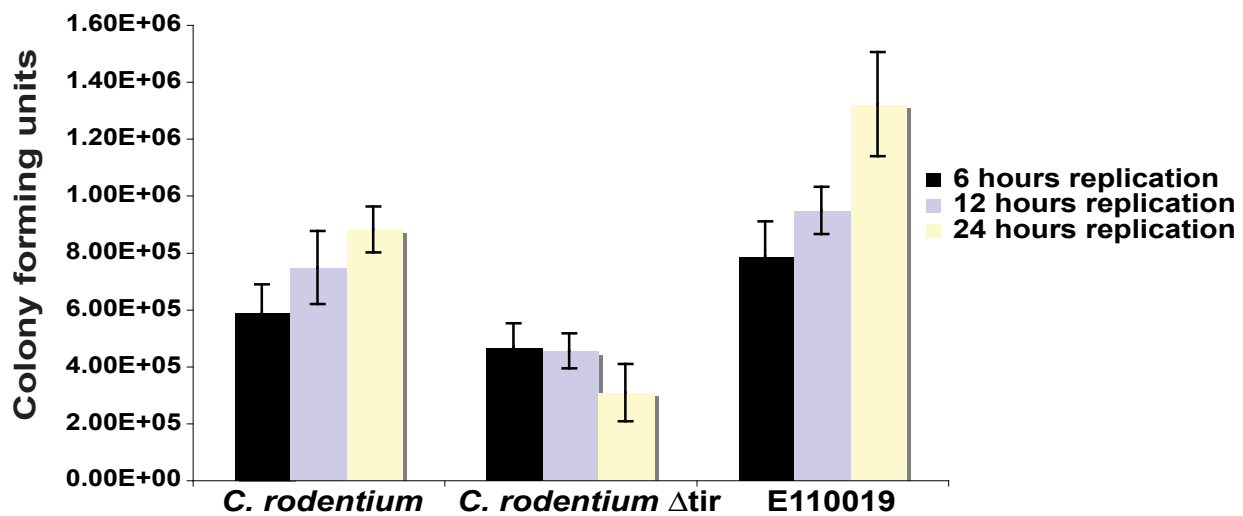

Supplement: Figure S8 — (A) Internalized EPEC survive and replicate in epithelial cells. HeLa cells were infected with E110019 for 30 min before the cells were washed with gentamycin to eliminate non invasive-bacteria. The cells were then incubated for 2, 8, 16 and 24 h in the presence of gentamycin. Cells were processed for immuno-fluorescence microscopy, bacteria were detected with Dapi (Dlue) and actin was labeled with phalliodin (Red). There was a time dependent increase in the level of intracellular bacteria. (B) Quantitative gentamycin protection assay of intracellular growth. HeLa cells were infected for 3 h with C. rodentium, C. rodentium Δtir and E110019 before extracellualr bacteria were eliminated with gentamycin. Cells were then incubated in the presence of gentamycin for 6, 12 or 24 h before the cells were lysed and plated for CFU counting. Results are representative of 3 independent experiments and are presents as mean±SEM. (1.92 MB PDF) [file ppat.1000683.s008.pdf]
